# Noninvasive biophotonic imaging for studies of infectious disease

**DOI:** 10.1111/j.1574-6976.2010.00252.x

**Published:** 2010-10-19

**Authors:** Nuria Andreu, Andrea Zelmer, Siouxsie Wiles

**Affiliations:** 1 Department of Medicine, Imperial College LondonLondon, UK; 2 Department of Infectious and Tropical Diseases, London School of Hygiene and Tropical MedicineLondon, UK; 3 Department of Molecular Medicine and Pathology, University of AucklandAuckland, New Zealand

**Keywords:** biophotonic imaging, infectious disease, bioluminescence, fluorescence, *in vivo*, luciferase, infection

## Abstract

According to World Health Organization estimates, infectious organisms are responsible for approximately one in four deaths worldwide. Animal models play an essential role in the development of vaccines and therapeutic agents but large numbers of animals are required to obtain quantitative microbiological data by tissue sampling. Biophotonic imaging (BPI) is a highly sensitive, nontoxic technique based on the detection of visible light, produced by luciferase-catalysed reactions (bioluminescence) or by excitation of fluorescent molecules, using sensitive photon detectors. The development of bioluminescent/fluorescent microorganisms therefore allows the real-time noninvasive detection of microorganisms within intact living animals. Multiple imaging of the same animal throughout an experiment allows disease progression to be followed with extreme accuracy, reducing the number of animals required to yield statistically meaningful data. In the study of infectious disease, the use of BPI is becoming widespread due to the novel insights it can provide into established models, as well as the impact of the technique on two of the guiding principles of using animals in research, namely reduction and refinement. Here, we review the technology of BPI, from the instrumentation through to the generation of a photonic signal, and illustrate how the technique is shedding light on infection dynamics *in vivo*.

## Introduction

Light is defined as electromagnetic radiation, particularly of wavelengths visible to the human eye (approximately 400–700 nm), that exists as tiny ‘packets’ called photons. Interestingly, light exhibits the properties of both particles and waves and when it propagates through tissue, undergoes a range of interactions depending on the structural arrangement and physical properties of the microenvironment. Such interactions have led to the development of the field of optical imaging, which encompasses a wide variety of methods and approaches (Table 1) such as visualizing tissue anatomy on the microscopic scale using the properties of light absorption and scattering (Zonios *et al.*, 2001), the rapidly evolving field of live cell fluorescence microscopy (Hoppe *et al.*, 2009), intravital microscopy in which the field of interest is located under a surgically implanted window (Helmchen & Denk, 2005) and the noninvasive localization and quantification of a photonic signal three-dimensionally in whole animals [e.g. by fluorescence molecular tomography (FMT); Ntziachristos, 2006].

**Table 1:** Optical imaging methodologies

Resolution	Technique	Contrast	Depth
Microscopic	Epi-microscopy	A, Fl	20 μm
	Confocal microscopy	Fl	500 μm
	Multiphoton microscopy	Fl	800 μm
Mesoscopic	Optical projection tomography	A, Fl	15 mm
	Optical coherence tomography	S	2 mm
	Laser speckle imaging	S	1 mm
Macroscopic	Hyperspectral imaging	A, S, Fl	<5 mm
	Endoscopy	A, S, Fl	<5 mm
	Fluorescence reflectance imaging (FRI)	A, Fl	<7 mm
	Diffuse optical tomography (DOT)	A, Fl	<20 cm
	Fluorescence resonance imaging (FRI)	A, Fl	<7 mm
	Fluorescence molecular tomography (FMT)	Fl	<20 cm
	Biophotonic Imaging (BPI)	Fl, E	<3 cm

Adapted from Weissleder & Ntziachristos (2003).

A, absorption; Fl, fluorescence; S, scattering; E, emission.

**Fig. 1:**
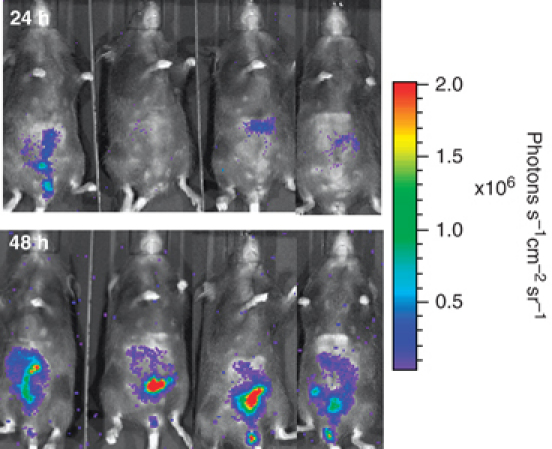
Monitoring transmission of the gastrointestinal pathogen *Citrobacter rodentium* through the faecal–oral route using BPI. Mice were exposed to infectious *C. rodentium* ICC180 in the cage environment and transmission and infection dynamics were determined by BPI. Images were acquired using an IVIS system (Caliper Life Sciences) with an integration time of 1 min and are displayed as pseudocolour images of peak bioluminescence, with variations in colour representing light intensity at a given location. Red represents the most intense light emission, while blue corresponds to the weakest signal. The colour bar indicates relative signal intensity (as photons s^−1^ cm^−2^ sr^−1^). The same four mice were imaged 24 h (top panel) and 48 h (bottom panel) after introduction into the contaminated cage.

Within the field of optical imaging, biophotonic imaging (BPI) is a highly sensitive noninvasive, nontoxic technique based on the detection of visible light that arises from either the excitation of a fluorescent protein (FP) or molecule or from an enzyme-catalysed oxidation reaction (a phenomenon known as bioluminescence). Although the light emitted may be dim, it is detectable externally using sensitive photon detectors such as those based on cooled, or intensified, charge coupled device (CCD) cameras, mounted within light-tight specimen chambers. As light passes through a range of tissue types (including skin, muscle and bone), it is possible to observe and quantify the spatial and temporal distribution of light production from within living animals (Fig. 1). While researchers typically use commercially available imaging systems (Table 2), some protocols are available for those with a more do-it-yourself approach or limited budget (Zacharakis *et al.*, 2005a,b; Hoffman & Yang, 2006; Hoffman & Zhao, 2006;).

**Table 2:** Commercially available BPI instrumentation

Manufacturer	Instruments	Features	Specifications
Berthold Technologies (http://www.bertholdtech.com)	NightOwl(2 camera options)	Bioluminescence Fluorescence	Various filters (340–1100 nm); tungsten halogen excitation source.
Biospace Lab (http://www.biospacelab.com)	PhotonImager	Bioluminescence Fluorescence Macrolens to convert to bioluminescence microscope Image freely moving animals (In Actio®)	Excitation filters span 400–800 nm; 6 emission filters; 150 W halogen excitation source
Caliper Life Sciences (http://www.caliperls.com)	IVIS (various models)	Bioluminescence Fluorescence Digital X-ray (Lumina XR) Image freely moving animals (Kinetic)	Excitation filters span 425–760 nm; Various options for emission filters spanning 500–875 nm; software for 3D reconstruction using spectral scanning (not all models).
Cambridge Research & Intrumentation (CRi) http://www.cri-inc.com	Maestro	Fluorescence Spectral scanning	Liquid crystal tunable filter allows spectral scanning over range of 500–950 nm in user-defined steps as small as 2 nm; xenon excitation source
Carestream Health (http://www.carestreamhealth.com)	Kodak *In Vivo* Imaging systems (various models)	Bioluminescence Fluorescence Digital X-ray (FX/FX-Pro)	Up to 28 excitation filters; 6 emission filters; 175 W xenon excitation source
Li-Cor Biosciences (http://www.licor.com)	Pearl (1 mouse); Odyssey® Imager+Mousepod™ (3 mice)	Near infra-red fluorescence	Two-channel laser excitation (excitation/emission filters): 685/720 nm and 785/820 nm
VisEn (http://www.visenmedical.com)	FMT 2500 Imaging system	Near infra-red fluorescence Two modes: Reflectance Imaging and Quantitative Tomography Multimodality adaptors for CT/MR/PET	Two channel laser excitation (excitation/emission filters): 670/700 nm and 745/780 nm
UVP (http://www.uvp.com)	iBox® Scientia Small Animal Imaging System(2 camera options)	Bioluminescence Fluorescence	Eight excitation filter positions; three emission filters: 515–570 nm, 485–655 nm, 570–640 nm;150 W excitation source

Upon contact with a host organism, pathogenic microorganisms utilize a wide variety of strategies to subvert host cell functions and modulate the immune response. Naturally, we wish to understand these strategies and develop interventions to circumvent them. Optical imaging techniques are at the forefront of such investigations *in vitro*. For example, the use of live cell microscopy is beginning to unravel the localized and transient interactions between eukaryotic cells and pathogenic microorganisms at the molecular level (Hoppe *et al.*, 2009). While possessing some limitations (Wiles *et al.*, 2006a), deliberately induced infections in well-defined animal models provide much useful information about disease processes in an approximation of their natural context *in vivo*. The use of animals is accompanied by ethical responsibilities and many countries promote the three Rs: replacement, reduction and refinement. As the name suggests, replacement refers to methods that avoid or replace the use of animals and include utilizing computer modelling, established human and animal cell lines and invertebrate models such as the fruitfly and nematode. Reduction refers to methods that minimize animal use and enable researchers to obtain comparable levels of information from fewer animals or to obtain more information from the same number of animals, thereby reducing the future use of animals. Refinement refers to improvements to scientific procedures and husbandry, which minimize actual or potential pain, suffering, distress or lasting harm and/or improve animal welfare.

**Fig. 2:**
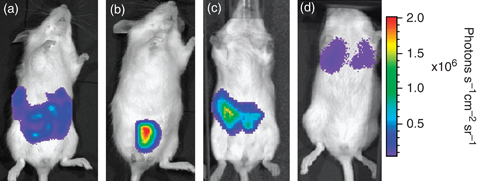
Monitoring correct dosing using BPI. Mice were inoculated with luminescent *Citrobacter rodentium* ICC180 via the intraperitoneal route (a, b) and by oral gavage (c, d), and the success of the administration was determined by BPI. The animals in (a) and (c) have been correctly dosed with administration of the inocula delivered into the peritoneum (a) and gastrointestinal tract (c). The animals in panels (b) and (d) have been incorrectly dosed, with the inocula being inadvertently delivered into the bladder (b) and lungs (d). Images were acquired using an IVIS system (Caliper Life Sciences) with an integration time of 1 min and are displayed as pseudocolour images of peak bioluminescence, with variations in colour representing light intensity at a given location. Red represents the most intense light emission, while blue corresponds to the weakest signal. The colour bar indicates relative signal intensity (as photons s^−1^ cm^−2^ sr^−1^).

BPI is a very powerful tool for implementation of two of the three Rs: refinement and reduction. Using traditional disease models, infected animals (often 3–10) are sacrificed at defined time points and tissues are excised for determination of pathogen numbers and localization. For example, a six time point experiment would result in the use of 18–60 animals. In contrast, the nondestructive nature of BPI allows the course of an infection to be monitored simply by imaging the photonic signal detected from within the same group of animals, typically six to eight in total. Importantly, multiple imaging of the same animal throughout an experiment allows disease progression to be followed with extreme accuracy, while allowing each animal to act as its own control. Furthermore, when constitutively expressed, bioluminescence is related to microbial numbers and can therefore be used for quantification of pathogen burden (Francis *et al.*, 2001; Rocchetta *et al.*, 2001; Wiles *et al.*, 2004; Rajashekara *et al.*, 2005;). This can result in significant refinements to *in vivo* models of infectious disease. For example, in a number of models, death of the animal results from the rapid and uncontrolled expansion of the infecting microorganism. With BPI, the photonic signal can be used to estimate whether an animal will survive or die, allowing for humane euthanasia perhaps even before the onset of clinical symptoms. For instance, the appearance of a signal in the cervical lymph nodes of mice exposed to spores of luminescent *Bacillus anthracis* can take from 2 to 4 days, but it is an unequivocal sign of a failure in the host innate immune response that leads to dissemination and death (Loving *et al.*, 2009). In addition, BPI can also result in a reduction in the levels of stress and/or discomfort experienced by experimental animals by avoiding the need for invasive sampling procedures routinely used to determine the bacterial load in specific tissues or fluids such as blood or the cerebrospinal fluid (Kadurugamuwa *et al.*, 2005c). Finally, BPI can provide real-time data on the effectiveness of the inoculation method (Kadurugamuwa *et al.*, 2005c; Glomski *et al.*, 2007b; Wiles *et al.*, 2007; Wiles et al., 2007). As a result, errors in administration can be detected immediately (Fig. 2) and animals can be eliminated from further study – thus minimizing any potential pain, suffering and distress for the animal and reducing variation by removing flawed scientific data.

## Factors influencing the sensitivity of BPI

While there are a number of factors that influence the sensitivity of BPI (Table 3), the main considerations are (1) the inherent features relating to the propagation of light through tissue, (2) the inherent background signals within living animals and (3) the availability of oxygen.

**Table 3:** Factors affecting the sensitivity of BPI

Sensitivity of the detection system
Level of fluorophore/luciferase expression
Wavelength of light emitted
Excitation wavelength (fluorescence)
Availability of cofactors
Location of the signal within the animal (tissue type and depth)
Whether the animal has fur or is pigmented
Background fluorescence/luminescence

### Properties of light propagation through tissue

The major challenges in optical imaging relate to the nature of the interactions between light and matter, namely scattering and absorption. Upon contact with tissue, photons in the visible and infrared wavelengths are highly scattered, resulting in photon diffusion; that is, photons do not propagate along straight lines but follow diffusive patterns. Furthermore, the intensity of light is reduced after passing through tissue. As a general rule, there is an approximate 10-fold loss of photon intensity for each centimetre of tissue depth (Contag *et al.*, 1995). This absorption demonstrates a characteristic spectral signature originating from endogenous chromophores. Within the visible spectrum (400–760 nm), haemoglobin is the primary chromophore that absorbs light within tissues (Taroni *et al.*, 2003). Haemoglobin absorbs in the blue and green part of the visible spectrum, but its absorption of wavelengths longer than 600 nm is reduced, allowing transmission of red light through several centimetres of tissue (Rice *et al.*, 2001). Melanin is also a significant contributor to absorption in pigmented animals, meaning that signals within animals with dark fur will be much more attenuated than signals from within nude animals or those with white fur. Shaving with clippers or removing fur with a depilatory agent can go some way towards minimizing this problem.

These concepts can be easily demonstrated by holding a flashlight behind your hand and observing the light emerging through your fingers (Doyle *et al.*, 2004). While light can be observed to pass through the skin, muscle and bone, clear images of the bones are not apparent due to the scattering of photons as they bounce through the tissue. Moreover, the light coming through the fingers is red. This is due to the greater absorption of the shorter wavelengths of light (blue and green) compared with the longer wavelengths (red light). While the spectral properties of the photonic signal as well as its location within the body will therefore have a significant impact on how much of the light is transmitted to the surface, the optical properties of tissues are predictable and can be modelled mathematically (Cheong *et al.*, 1990; Rice *et al.*, 2001; Ripoll & Ntziachristos, 2003; Ripoll *et al.*, 2003;), allowing a certain degree of quantification and resolution.

### Background fluorescence/luminescence

In general, luminescence imaging is much more sensitive than fluorescence imaging as a result of better signal-to-noise ratios. This is mainly due to the high levels of background fluorescence *in vivo* compared with luminescence (Troy *et al.*, 2004). Indeed, tissue autofluorescence is one of the major causes for concern in imaging fluorescence, and is due to endogenously produced fluorophores such as keratin, porphyrins, NAD(P)H, collagen and elastin. Generally, autofluorescence from most materials, including tissue, is higher at short wavelengths and decreases in the red (Troy *et al.*, 2004). Although the level of autofluorescence is dependent on the intensity and wavelength of the excitation source, autofluorescence is generally many orders of magnitude brighter than autoluminescence. Autofluorescence in the green and red-orange spectral regions is fairly uniform over the entire animal. For the far-red and near-infrared (NIR) spectral regions, tissue autofluorescence is more concentrated in the intestinal area due to the presence of chlorophyll in rodent diet (Troy *et al.*, 2004). A number of alternative diets are available, including an alfalfa-free diet and a purified diet (containing predominantly cornstarch and milk casein), both of which drastically reduce the levels of background fluorescence in the red and NIR parts of the spectrum in the abdominal region (Inoue *et al.*, 2008). In addition, advances in spectral unmixing algorithms have improved the signal-to-noise ratio of fluorescent signals by separating the specific signal from the autofluorescence.

### Oxygen requirement

Oxygen is a cofactor required by all luciferases discovered to date. However, it has been reported that luminescence can be detected from marine bioluminescent bacteria under oxygen concentrations as low as 10 nM (Bourgois *et al.*, 2001). Furthermore, chromophore maturation in almost all FPs requires molecular oxygen, but is prevented only by rigorously anoxic conditions (<0.75 μM O_2_), and is readily detected at 3 μM O_2_ (Hansen *et al.*, 2001). As a result of the reliance upon oxygen for generation of a photonic signal, it has been suggested that BPI may be of limited use in anaerobic environments, such as the necrotic cores of large tumours. While this is certainly an important consideration, BPI can actually shed some light on how anaerobic these environments are. For example, the presence of ‘strict’ anaerobes such as *Bacteroides* residing within the gastrointestinal tract has led to the long-held belief that this environment is anaerobic. In fact, the tissues surrounding the lumen of the gastrointestinal tract are oxygen rich, and oxygen has been shown to diffuse into the intestine (He *et al.*, 1999). BPI has clearly demonstrated that this level of oxygen is sufficient to allow the generation of detectable light by a luminescent derivative of the enteric pathogen *Citrobacter rodentium* colonizing the murine colon and caecum (Wiles *et al.*, 2006b). Furthermore, light production was only seen in live animals, suggesting the requirement for a circulating blood supply to provide sufficient oxygen. A similar observation has been reported for *Salmonella*, with luminescence in the caecum ceasing after cervical dislocation of the animal (Contag *et al.*, 1995), while Lane *et al.* (2007) reported a waning of the bioluminescent signal shortly after dissection for the bladder, and mincing of the kidneys was necessary to replicate by *ex vivo* imaging the signal observed *in vivo*. These diverse observations could be related to variations in the oxygen concentrations in different *in vivo* niches, as well as the inherent characteristics of the microorganisms being studied. Importantly, it has been demonstrated that aerobic respiration is required for commensal and pathogenic *Escherichia coli* to colonize mice (Jones *et al.*, 2007) and that many species of *Bacteroides* can grow in nanomolar concentrations of oxygen (Baughn & Malamy, 2004). Indeed, homologues of cytochrome *bd* oxidase (CydA), essential for oxygen consumption, have been identified in the genomes of many prokaryotes classified as strict anaerobes (Baughn & Malamy, 2004). This has led to the suggestion of a new term, nanaerobes, for such organisms that can benefit from, yet do not require, oxygen for growth. Perhaps a number of the environments previously thought to be anaerobic are nanaerobic.

## Performing BPI

There are two main techniques for performing BPI: planar imaging and tomographic imaging. Planar imaging is the simplest method, being easy to implement and offering high throughput. However, it does have limitations, most notably the nonlinear relationship between the signal detected, its location within the animal (depth) and the optical properties of the surrounding tissues. In contrast, tomographic imaging enables quantitative three-dimensional volumetric imaging but is more time-consuming and labour-intensive.

### Planar imaging

Typically, a photographic reference image is first acquired under weak illumination. In fluorescence imaging, this is followed by illumination of the subject, usually with a broad light beam passing through a filter tuned to the excitation wavelength of the fluorophore of interest. Typically the light source is located on the same side as the detector (known as epi-illumination) but the light source may also be located on the opposite side to the detector (known as trans-illumination). In general, trans-illumination is capable of imaging signals located deeper within the tissue. The resulting biophotonic signal is then captured in complete darkness, which may take from seconds to minutes depending on the strength and location of the signal and the sensitivity of the imaging system. Again, the emitted light can be captured using particular bandwidth emission filters. CCD cameras spatially encode the intensity of incident photons, which are then shown as a pseudocolour image superimposed on the grey-scale photographic image. Bioluminescent signals are detected in the same manner but without the second illumination step. Typically, data are quantified by region-of-interest analysis, measuring absorption units or efficiency (the fraction of fluorescent photons relative to each incident excitation photon) for fluorescence and photon flux for bioluminescence.

Importantly, the properties of tissue attenuation previously discussed mean that images are surface-weighted; light sources closer to the surface of the animal appear brighter compared with those in deeper tissue, highlighting the need for further information regarding signal localization. This may take the form of pilot experiments in which tissues are harvested after imaging to determine the location of the photonic signal. Hillman & Moore (2007) developed a novel system for aiding localization, which they termed dynamic fluorescence molecular imaging. The technique involves acquiring a series of dynamic (time-sequence) images following a tail-vein injection of an NIR dye. As the dye circulates throughout the body, each organ displays characteristic and visible pharmacokinetics. This system is now marketed by CRi (http://www.cri-inc.com) as Dynamic Contrast Enhancement (DyCE) for use with the Maestro imaging system (Table 2). Essentially, the system resolves the data using a series of algorithms and displays the organs in pseudocolour; for example, the brain may appear as blue, the liver as red, while the kidneys in purple.

Advanced understanding of the depth-dependent attenuation of light at different wavelengths and the development of mathematical models now allows the generation of a three-dimensional reconstruction of bioluminescent/fluorescent sources from a series of planar images. For example, diffuse luminescence tomography is based on the acquisition of a photographic image, followed by a structured light image to reconstruct the tomography of the surface of the subject. A number of images are then acquired using two or more narrow band-width emission filters and the data are combined to produce a high-resolution map of the photon density at the surface. The reconstruction algorithm then consists of finding an approximate solution to a system of linear equations that relate the source strength at each point inside the object to the photon density at the surface (Kuo *et al.*, 2007).

### Tomographic imaging

True tomographic imaging has mainly been applied to imaging fluorescence (also referred to as FMT), and involves the illumination of the sample at different points or projections and the collection of the emitted photonic signal using various photodetector sets or a CCD camera. As with planar imaging, the emitted light can be captured using particular bandwidth emission filters. There are three distinct methods by which the tissue can be illuminated: using light of constant intensity [termed constant wave (CW)], using light of modulated wavelength [termed frequency domain (FD)] or using ultrafast (femtosecond to picosecond) photon pulses and resolving the arrival of the photons as a function of time [termed time-domain (TD)]. Each method has distinct advantages and disadvantages, and selection largely depends on the specific application (Ntziachristos *et al.*, 2005). Importantly, each source–detector pair effectively implements a different projection through the tissue and this is combined with mathematical formulae that describe photon propagation in tissues as well as algorithms for image reconstruction. Increasing the number of source–detector pairs increases the accuracy of the reconstructed image. Recently, Turner *et al.* (2005) reported the rotation of an object of interest in front of the illumination path, using a CCD camera to collect up to 72 projections. Termed complete projection tomography, the authors demonstrated the ability to resolve both the location and size of the photonic signal. More comprehensive descriptions of FMT can be found in a number of recent reviews (Ntziachristos *et al.*, 2005; Ntziachristos, 2006;).

Performing such tomographic analyses of bioluminescent sources can be carried out in a similar fashion but in the absence of external illumination (Gu *et al.*, 2004; Wang *et al.*, 2004a;). However, the lack of external illumination makes it mathematically more difficult to resolve the photonic signal as there are fewer projections (source–detector pairs) available. Furthermore, as the bioluminescent signal is continuously on during the measurement, bioluminescence tomography operates in CW mode only. For these reasons, as well as the recent withdrawal of the only commercially available system capable of rotating the animal and capturing multiple projections, such tomographic reconstruction of bioluminescent sources is rare.

## Animal welfare during BPI

To perform BPI, animals are most often anaesthetized for restraint purposes, using either gaseous or injectable anaesthetic agents. Furthermore, if generation of a photonic signal is dependent upon the addition of exogenous substrate, this must be administered by an appropriate route. The two main implications for animal welfare therefore relate to anaesthesia and the number and frequency of injections.

### Anaesthesia

There are a number of factors that will influence the type of anaesthetic agent selected for BPI, including the animal species and strain, the time required to remain under anaesthesia and the equipment available. Both the type of imaging being performed (planar vs. tomographic) as well as the level of photonic signal will determine the period of time required for the animals to remain under anaesthesia, usually in the range of 1–30 min. As mice cannot regulate their own body temperature under anaesthesia, steps should be taken to maintain their core temperature both during imaging and until full recovery. In most commercial imaging systems, the shelf of the imaging chamber is heated for exactly this reason. It is preferable to anaesthetize mice using inhalational agents such as isoflurane, as the depth and duration can be more easily controlled and standardized. Inhaled agents are mainly eliminated by the lungs, whereas injectable agents need to be metabolized by the liver and excreted by the kidneys, a process that can be prolonged. Recovery is therefore more rapid from inhaled agents, which is important in regaining normal physiology, to control postprocedural hypothermia and fluid or electrolyte imbalance. Inhalational agents are also suitable for high-frequency anaesthesia studies, where animals are repeatedly imaged. For example, mice can be imaged three to four times a day using isoflurane although ideally this intensive monitoring regime would not be followed for more than 3 days. Where injectable agents are used, each animal should be weighed and dosed according to its bodyweight. Ketamine can cause muscle rigidity, and so in certain situations the mice may appear to twitch. This is less than ideal, especially if the photonic signal is located in the limbs. Anaesthetized animals must be monitored to ensure that they remain in the proper anaesthetic plane. The animals should not be too lightly anesthetized that they regain consciousness, or too deep that vital functions are compromised. For prolonged periods of anaesthesia (>30 min), it is recommended to use an ophthalmic artificial tear ointment such as Lacrilube (Allergan, Buckinghamshire, UK) to prevent corneal drying and trauma.

### Number and frequency of injections

In accordance with the ethical responsibilities placed on researchers using animals, there are published good practice guidelines on the total number and frequency of injections that can be administered to an animal (Diehl *et al.*, 2001). For BPI, this limits the number of imaging sessions that can be performed on an animal throughout an experiment, particularly if injectable anaesthetic agents and substrate are administered. Suggested maximum doses and frequencies for mice are given in Table 4.

**Table 4:** Suggested maximum volumes and frequencies of administration of substances to mice (in accordance with Diehl *et al.*, 2001)

	IP	IM	SC	IV
Maximum number of doses	24	6	24	14
Maximum daily volume	20 mL kg^−1^	500 μL	20 mL kg^−1^	10 mL kg^−1^
Number of daily doses <7 days	2–3	2	3	1–2*
Number of daily doses >7 days	1	1	2	<1*

* For intravenous administration, 1 dose per day should be administered for no more than 6 days, while 2 doses per day should be administered for no more than 2 days.

IP, intraperitoneal; IM, intramuscular; SC, subcutaneous; IV, intravenous.

## Generation of a photonic signal for BPI

As stated previously, the biophotonic signal detected during BPI can be either bioluminescent or fluorescent. In this section, we describe the basic properties of these two very different phenomena.

### Bioluminescence

Bioluminescence is widely distributed in nature, occurring in a remarkably diverse set of organisms, including bacteria, dinoflagellates, fungi, fish, insects, shrimp and squid. Bioluminescence arises from the oxidation of a substrate (a luciferin) by an enzyme (a luciferase), which usually requires energy (in the form of FMNH_2_ and ATP) and oxygen. Luciferin and luciferase are generic terms as none of the major classes share sequence homology. While phylogenetic analyses suggest that bioluminescence has had more than 30 independent origins, there are five basic luciferin–luciferase systems. Most widely studied of the bioluminescence systems are those belonging to luminous beetles in the family Lampyridae (the most studied being the firefly *Photinus pyralis* and the click beetle *Pyrophorus plagiophtalamus*), the sea pansy *Renilla reniformis*, the marine copepod *Gaussia princeps* and numerous luminous bacteria (terrestrial *Photorhabdus luminescens* and marine *Vibrio* and *Photobacterium* sp.).

The beetle luminescence reaction is catalysed by a monomeric luciferase of approximately 62 kDa encoded by a single gene (*luc*) and involves the oxidation of a benzothiazoyl-thiazole ‘luciferin’ (commonly referred to as luciferin) and ATP, resulting in the production of oxyluciferin, AMP, CO_2_ and the emission of light. In *P. pyralis* this light has a peak at 560 nm (Hastings, 1996) while *P. plagiophtalamus* emits light of distinct peaks, ranging from 546 to 593 nm (Wood *et al.*, 1989). Interestingly, the light generated by the firefly luciferase is influenced by temperature, shifting to a peak of 610 nm at 37 °C (Zhao *et al.*, 2005a). The firefly luciferase catalyses the most efficient bioluminescent reaction known (i.e. the amount of light generated in relation to the energy expended) with a quantum efficiency of 0.41 (Ando *et al.*, 2007) and tends to be the reporter of choice for expression by eukaryotic cells. The genes required for luciferin production have not been completely elucidated and therefore exogenous luciferin must be administered by an appropriate route, most commonly via intraperitoneal injection, a few minutes before imaging. Fortunately, at the doses administered, luciferin does not appear to be toxic to animals and rapidly distributes throughout the mouse (Contag *et al.*, 1997), crossing the blood–brain and placental barriers (Lipshutz *et al.*, 2001; Rehemtulla *et al.*, 2002;). Furthermore, as it is given in excess, substrate availability is not generally considered to be a limiting factor. Recently, it has been suggested that if luciferin is not required to be distributed throughout the animal, it can be directly injected into specific sites of interest instead. Researchers have utilized this delivery method to image luciferase expression in muscle, the knee joint (Bloquel *et al.*, 2006) and the vaginal tract (Doyle *et al.*, 2006a). Furthermore, Buckley *et al.* (2008) demonstrated that when imaging in the nasal and pulmonary airways of mice, compared with intraperitoneal injection, intranasal instillation yields about a 10-fold increase in sensitivity with an approximate 30-fold reduction in luciferin usage. Alternative methods of luciferin delivery have been described in the literature and include the use of an osmotic pump for continuous delivery (Gross *et al.*, 2007), encapsulation of the luciferin within long circulating liposomes (Kheirolomoom *et al.*, 2010) or within food and water (Hiler *et al.*, 2006).

The monomeric sea pansy and copepod luciferases, encoded by the genes *rluc* (also referred to as *ruc*) and *gluc*, respectively, are members of the most common natural bioluminescence system, deep-sea imidazolopyrazine bioluminescence, which has been reported in seven phyla and approximately 90 genera (Thomson *et al.*, 1997). Coelenterazine is an imidazolopyrazine derivative that acts as the luciferin that, when oxidized by the appropriate luciferase, produces carbon dioxide, coelenteramide and light in the blue part of the spectrum (480 nm) (Shimomura *et al.*, 1978; Shimomura & Teranishi, 2000;). Interestingly, Gluc is strongly resistant to heat and extreme pH (Wiles *et al.*, 2005b), and has been shown to be secreted from both mammalian and bacterial cells (Tannous *et al.*, 2005; Andreu *et al.*, 2010;). However, the requirement for coelenterazine is an important limiting factor in using Gluc and Rluc. As with luciferin, coelenterazine has to be administered by an appropriate route, most often by tail-vein injection. In contrast to luciferin, the bioavailability of coelenterazine is limited *in vivo*, at least in part by the multidrug resistance *P*-glycoprotein (MDR1), which transports coelenterazine and similarly structured compounds out of the membranes of mammalian cells (Pichler *et al.*, 2004). Furthermore, coelenterazine is highly chemiluminescent, undergoing luciferase-independent oxidation (Shimomura & Teranishi, 2000), thus limiting sensitivity by reducing the signal-to-noise ratio. Indeed, we have been unable to distinguish *in vivo* the signal from Gluc-expressing *Mycobacterium smegmatis* from the strong background produced by the coelenterazine substrate alone (Andreu *et al.*, 2010). Nevertheless, BPI of tumour cells expressing Gluc and Rluc has been reported (Bhaumik & Gambhir, 2002; Tannous *et al.*, 2005;).

The bacterial luminescence reaction involves the oxidation of a long-chain aldehyde and reduced flavin mononucleotide (FMNH_2_) resulting in the production of oxidized flavin (FMN), a long-chain fatty acid and light at 490 nm (Hastings & Presswood, 1978; Baldwin *et al.*, 1984; Campbell, 1989;). The reaction is catalysed by bacterial luciferase, a heterodimeric enzyme of 77 kDa composed of an α and a β subunit encoded by the *luxA* and *luxB* genes, respectively. The *luxC, D* and *E* genes encode the subunits of a multienzyme complex responsible for regeneration of the aldehyde substrate from the fatty acid produced by the reaction. A significant advantage of the bacterial luciferase system is therefore the ability to express the biosynthetic enzymes for substrate synthesis.

### Fluorescence

The phenomenon of fluorescence was first described in 1845 by John Frederick William Herschel, who observed a superficial blue glow in a solution of quinine in the sunlight (Herschel, 1845). The intervening years have now seen FPs and probes used ubiquitously in biological research. Irradiation of a fluorescent compound with light of a suitable wavelength leads to the transition of an electron in the molecule to a higher energy state (excitation). This process is almost instantaneous, taking around 10^−15^ s. Upon return of the electron to a lower energy level (around 10 ns), light of lower energy is emitted, giving the fluorescent signal. Because lower energy light is emitted, it is red-shifted in the spectrum when compared with the excitation light, a phenomenon known as the Stokes shift.

#### FPs

Green fluorescent protein (GFP) from the jelly fish *Aequorea victoria* was first described in 1962 by Osamu Shimomura (Shimomura *et al.*, 1962; Shimomura, 2005;) who was awarded the 2009 Nobel Prize in Chemistry for his discovery, together with Roger Tsien and Martin Chalfie. In the 15 years since Chalfie first reported the use of GFP as a marker of gene expression in the nematode *Caenorhabditis elegans* (Chalfie *et al.*, 1994), numerous FPs in all colours of the rainbow have been discovered and developed, many of them in Roger Tsien's lab. Most FPs derived from GFP have emission peaks in the blue, green or yellow range of the spectrum. To date, the only exception is R10-3, a dual emitting FP with emission peaks at 555 and 585 nm (Mishin *et al.*, 2008). Red FPs are often based on proteins from other sea organisms, such as DsRed from the coral *Discosoma* sp. The FP family is constantly expanding; over 40 coral FPs were described recently by Alieva *et al.* (2008), which range in colour from cyan to chromored, while the ‘mFruit’ variants of red FPs were developed by Shaner *et al.* (2004).

For a fluorescent marker to be successfully used for BPI, it has to fulfil several criteria including suitable excitation and emission wavelengths, photostability (a measure of the time an FP takes to lose 50% of its initial emission, under constant illumination), brightness and maturation speed (the time taken to full chromophore formation). The brightness of an FP is proportional to the product of its quantum yield and extinction coefficient. The quantum yield is the ratio of the number of emitted photons to absorbed photons, while the molar extinction coefficient describes the amount of light absorbed by a 1M solution of the protein at a path-length of 1 cm at a given wavelength (M^−1^ cm^−1^). Because light penetration of tissue depends heavily on wavelength, with longer wavelengths (ideally >650 nm) being more efficient (as discussed earlier), we focus on fluorescent molecules whose emission are in the red or the far red end of the spectrum, which are most suitable for BPI (Table 5). Excellent reviews on the topic of FPs in general can be found elsewhere (Shaner *et al.*, 2005, 2007; Remington, 2006; Pakhomov & Martynov, 2008).

**Table 5:** Characteristics of the fluorescent proteins deemed most suitable for BPI

Fluorescent protein	Excitation max.	Emission max.	Origin	Quantum yield	Excitation coefficient (M^−1^ cm^−1^)	Brightness (% DSRed)*	Maturation *t*_1/2_(min)	Reference
DsRed	558	583	*Discosoma* sp.	0.79	75 000	100	∼600	Shaner *et al.* (2004)
tdTomato	554	581	DsRed	0.69	138 000	160	60	Shaner *et al.* (2004)
mCherry	587	610	DsRed	0.22	72 000	27	15	Shaner *et al.* (2004)
mRaspberry	598	625	DsRed	0.15	86 000	22	55	Wang *et al.* (2004b)
mPlum	590	649	DsRed	0.1	41 000	7	100	Wang *et al.* (2004b)
TurboRFP	553	574	eqFP578	0.67	92 000	104	ND	Merzlyak *et al.* (2007)
TagRFP	555	584	eqFP578	0.48	100 000	81	100	Merzlyak *et al.* (2007)
Katushka	588	635	eqFP578	0.34	65 000	37	20	Shcherbo *et al.* (2007)
mKate	588	635	eqFP578	0.28	31 500	15	75	Merzlyak *et al.* (2007), Shcherbo *et al.* (2007)
tdKatushka2	588	633	eqFP578	0.37	132 500	82	ND	Shcherbo *et al.* (2009)
mKate2	588	633	eqFP578	0.4	62 500	42	<20	Shcherbo *et al.* (2009)
RFP611	559	611	eqFP611	0.48	120 000†	97†	110	Kredel *et al.* (2008)
					151 000‡	122‡		
RFP639	588	639	eqFP611	0.18	69.000†	21†	90	Kredel *et al.* (2008)
					110 400‡	34‡		
tdRFP611	558	609	eqFP611	0.47	70 000†	56†	225	Kredel *et al.* (2008)
					144 000‡	114‡		
tdRFP639	589	631	eqFP611	0.16	90 400†	24†	<480§	Kredel *et al.* (2008)
					110 000‡	30‡		
mRuby	558	605	eqFP611	0.35	112 000†	66	168	Kredel *et al.* (2009)
AQ143	595	655	aeCP597	0.04	90 000	6	ND	Shkrob *et al.* (2005)
IFP1.4	684	708	DrBphP	0.07	92 000	11	ND	Shu *et al.* (2009)

Key:

* Brightness is calculated as extinction coefficient × quantum yield.

† As determined by alkaline denaturation method.

‡ As determined by dynamic difference method.

§ As determined from expression in HEK293 cells; ND, not determined.

In the last few years, several groups of new red FPs have been reported. Based on DsRed, mRFP1 was the first monomeric red fluorescent protein (RFP) reported (Campbell *et al.*, 2002). This protein was then subjected to molecular-directed evolution, which resulted in several new FPs, ranging in colour from yellow to dark red (the ‘mFruits’; Shaner *et al.*, 2004). The most notable of these are tdTomato and mCherry. Even though the excitation and emission spectra for tdTomato (554 and 581 nm, respectively) are slightly lower than would be ideal for BPI, its exceptional brightness (160% of the original DsRed), fast maturation and high photostability make it a candidate worth considering. mCherry, the only protein in this study that emits light at a wavelength over 600 nm, is less bright (27% of DsRed) but similarly fast maturing and photostable. In our experiments, both proteins are well-expressed in *E. coli, M. smegmatis*, and *Mycobacterium tuberculosis* after codon optimization (Carroll *et al.*, 2010). Wang *et al.* (2004b) used iterative somatic hypermutation to create two further far-red-emitting monomeric FPs, mRaspberry and mPlum. In this elegant approach, the gene for mRFP1.2 was expressed in a human B cell line and mutants were selected for their brightness and red-shifted emission using fluorescence-activated cell sorting. As a result, mRaspberry displays excitation and emission maxima at 598 and 625 nm, respectively, with a maturation time similar to tdTomato. While the long excitation and emission wavelengths appear promising, this FP shows only 22% of the brightness of DsRed, and low photostability with 50% of emission intensity lost after only 14 s. Conversely, the furthest red-emitting protein in the mFruit series, mPlum (emission at 649 nm), takes 80 s to lose half its emission intensity, but displays <10% of the brightness of DsRed.

Another group of proteins emerged from two FPs isolated from the sea anemone *Entacmaea quadricolor*, eqFP578 and eqFP611 (Wiedenmann *et al.*, 2002; Merzlyak *et al.*, 2007; Shcherbo *et al.*, 2007, 2009; Kredel *et al.*, 2008, 2009). Both are characterized by relatively high brightness and a reduced tendency to oligomerize as compared with DsRed, which in its original configuration forms tetramers. Merzlyak *et al.* (2007) used the dimer-forming eqFP578 as a basis to perform random mutagenesis with the aim to optimize maturation time while maintaining brightness and a red colour. The resulting TurboRFP displays excitation and emission peaks of 553 and 574 nm, respectively, fast maturation at 37 °C, high pH stability, and brightness comparable to DsRed. Using site-directed mutagenesis, they then developed a monomeric version of TurboRFP called TagRFP. This protein has slightly red-shifted excitation and emission maxima (555 and 584 nm, respectively), but is somewhat less bright; a more photostable version (TagRFP-T) is also available (Shaner *et al.*, 2008). With the aim of developing a bright, far-red protein for whole-body imaging, Shcherbo *et al.* (2007) used a combination of site-specific and random mutagenesis to TurboRFP and several of its precursors. A library of ∼100 000 clones was screened for high brightness specifically at emission wavelengths of >650 nm. A red-shifted variant of TurboRFP was selected and further optimized, yielding the final product Katushka. At 635 nm, the emission of Katushka is further red shifted in comparison with mCherry, while the excitation peaks of the two proteins are very similar. Furthermore, Katushka matures faster, is more photostable, and is 1.4 times brighter than mCherry, making it an excellent candidate for BPI provided its dimeric state is tolerated by the host cell. Whole-body imaging of transgenic *Xenopus laevis* clearly showed superior tissue penetration of light when using Katushka; mPlum was also expressed but could hardly be detected, underlining the importance of brightness of an FP as well as wavelength characteristics. Because Katushka is dimeric, the authors also applied the mutations that gave rise to the monomeric protein TagRFP (Merzlyak *et al.*, 2007), resulting in mKate. This protein has spectral properties very similar to Katushka, but its maturation (half time of 75 min) and pH stability are inferior, although the brightness level is comparable to that of mCherry. In an effort to improve the brightness of far-red FPs, the same group recently reported mKate2, an enhanced version of mKate, and tdKatushka2, a tandem dimer version of Katushka (Shcherbo *et al.*, 2009), which display two- to almost threefold increased brightness over their respective precursors.

The second protein isolated from *E. quadricolor*, eqFP611 (Wiedenmann *et al.*, 2002), has given rise to a number of variants emitting in the far red, as described by Kredel *et al.* (2008, 2009). EqFP611 in its natural form is characterized by a far-red emission peak at 611 nm, a large Stokes shift of 52 nm and low aggregation tendency. However, this protein only folds efficiently at temperatures <30 °C and its use *in vivo* in mammalian systems is therefore limited. Using combined directed and random mutagenesis approaches, the researchers generated several variants of eqFP611, the two most interesting being RFP611 and RFP639, where the number indicates the emission maximum of each protein. Both proteins show good folding properties at 37 °C and high brightness. RFP611 has an excitation maximum of 559 nm and is 3.6 times brighter than mCherry, although its maturation time is slower at a half time of 1.83 h, and it is slightly less photostable (Kredel *et al.*, 2008). The excitation peak of RFP639 is at 588 nm, which makes its spectral properties similar to those of mCherry, with the brightness levels of both proteins being similar. RFP639 is approximately three times more photostable but has a longer maturation half time (1.5 vs. 0.25 h for mCherry). Because both RFP611 and RFP639 have a tendency to form tetramers, tandem-dimeric versions of each protein were described in the same study, with only slightly different spectral properties (Kredel *et al.*, 2008). mRuby, a monomeric version of RFP611, was recently presented and is characterized by excitation and emission peaks at 558 and 605 nm, respectively (Kredel *et al.*, 2009). It displays a fairly long maturation time (half time of 2.8 h) and is 1.5 times brighter than mCherry as measured by the authors, but is slightly less photostable. It is also extremely pH stable.

One FP that reaches the 650-nm barrier of emission is AQ143 (Shkrob *et al.*, 2005). This protein was derived from a blue nonfluorescent chromoprotein of the beadlet anemone *Actinia equine* (aeCP597) after random and site-specific mutagenesis. Its excitation and emission maxima are at 595 and 655 nm, respectively, which is the furthest red-shifted FP reported so far. However, like other far-red-emitting proteins (e.g. mPlum), it suffers from low brightness. Recently, Shu *et al.* (2009) demonstrated a new approach for engineering an infrared fluorescent protein for BPI using a truncated form (DrCBD) of the bacteriophytochrome DrBphP from *Deinococcus radiodurans*. The authors demonstrated that DrCBD, which consists of only the chromophore-binding domain, incorporates biliverdin, an intermediate in haeme catabolism produced by haeme oxygenase (HO-1), as the chromophore. The first variant, IFP1.0, was coexpressed with cyanobacterial HO-1 in *E. coli* and produced infrared fluorescence with an emission maximum at 722 nm. This protein was then subjected to mutagenesis and directed evolution, giving rise to further improved variants IFP1.1–IFP1.4, the latter of which is characterized by excitation and emission maxima of 684 and 708 nm, respectively, but suffers from low brightness similar to mPlum and other far-red proteins, and low photostability (50% bleaching after 8.4 s). However, after intravenous infection of mice with an adenovirus construct carrying the gene for either IFP1.1 or mKate, fluorescence from IFP1.1 could be readily detected in the livers of transfected mice after injection of the cofactor biliverdin. Low mKate fluorescence could also be detected, which was improved after removal of the overlying tissues (skin and peritoneum). Considering that mKate and IFP1.1 are of similar brightness, this once again demonstrates that tissue penetration and brightness both play important roles when choosing an appropriate fluorescent marker for BPI.

#### Alternatives to FPs

Although genetically encoded markers are very useful for imaging of preclinical animal models, their construction involves substantial time and resource commitments for each individual microorganism of interest, and they are not applicable in clinical settings. Leevy *et al.* (2006, 2008b) have suggested the use of an injectable NIR probe consisting of a bacterial affinity group conjugated to an NIR dye as an alternative. The researchers used a synthetic zinc(II) coordination complex [zinc(II)dipicolylamine or Zn-DPA], which targets the anionic surfaces of bacterial cells in general, linked to an NIR carbocyanine fluorophore with an excitation wavelength of 794 nm and emission wavelength of 810 nm; synthesis of this probe is described in Leevy *et al.* (2006). The authors demonstrated the ability of the probe to target *Staphylococcus aureus* in a mouse leg infection model, where 5 × 10^7^ bacteria were visualized by noninvasive *in vivo* imaging after intramuscular injection into the posterior leg and intravenous application of the NIR probe (Leevy *et al.*, 2008a). Even though this is a promising proof-of-principle study, the site of infection is isolated, shallow, well-separated from major organs, and displays low inherent background. Imaging of bacteria in deeper tissues with higher background fluorescence could, however, prove more difficult, and reduce sensitivity so that bacterial numbers of 10^8^ or more in a defined space would be needed, which only occurs in advanced stages of infection.

In recent years, quantum dots (QDs) have received some attention as probes for BPI, especially in cancer research (a short overview is given in Bentolila *et al.*, 2009). QDs are small fluorescent nanocrystals (usually a few tens of nanometers), which are made of inorganic semiconductor materials. They possess remarkable optical properties such as extremely high brightness and photostability, wide excitation and narrow emission spectra. What makes them most interesting for BPI is the fact that their emission wavelength depends on their size and can therefore be manufactured and fine-tuned as needed, from the UV to the infrared. However, using the previously described Zn-DPA bacterial affinity probe coupled to highly fluorescent QDs with an emission maximum of 800 nm via a biotin/streptavidin linker, Leevy *et al.* (2008a,b); found that the size of the QDs inhibited binding of the probe to its target on the bacterial surface and abolished staining capabilities of the probe in the case of *S. aureus* and two smooth strains of *E. coli*. Yet a rough mutant of *E. coli* could be successfully labelled, apparently owing to the lack of branched O-antigen components of the lipopolysaccharide on the cell surface, which prevent the Zn-DPA/QD complex from binding to its lipid A target deeper in the membrane. When 10^8^ prelabelled rough *E. coli* were injected into the hind leg of a nude mouse and the animal was imaged, the signal-to-noise ratio improved when compared with labelling with the Zn-DPA/NIR probe alone.

Even though autofluorescence is decreased with NIR emission wavelengths, tissue absorption and scattering still impede the amount of excitation light that reaches the fluorophore inside the tissue, particularly because QDs efficiently absorb blue light. To circumvent this problem, So *et al.* (2006a,b); have created so-called ‘self-illuminating’ QDs in which commercially available QDs were coupled to the *Renilla* luciferase (Luc8; Loening *et al.*, 2006). Once the luciferase is presented with its substrate coelenterazine, the produced broad-spectrum blue light is transferred to the QDs by bioluminescence resonance energy transfer, which in turn leads to emission of light by the QDs in the red to NIR part of the spectrum, depending on the QD used. By completely eliminating the need for excitation light, the signal-to-noise ratio was increased in some instances to >10^3^ after injection of the luc8/QD complex into nude mice. This very elegant approach could be refined by combining different luciferases with QDs of different wavelengths. If a reliable method can be found to specifically detect and label bacteria with QDs, this could be a very advantageous and efficient methodology for the *in vivo* imaging of bacterial infections.

## Developing bioluminescent/fluorescent microorganisms

The development of vectors for conferring a genetically encoded bioluminescent/fluorescent phenotype onto microorganisms allows the monitoring of any population provided the reporter genes are sufficiently and stably expressed and do not alter the ecological fitness and competitiveness of the host species. These issues should be addressed separately for each host/marker combination; genetic constructs should be optimized to achieve the highest possible expression levels without toxicity or effects on virulence.

### Stable reporter gene expression

Integration of the reporter gene onto the chromosome is favourable over expression from a plasmid to ensure stable and homogenous expression levels throughout the microbial population and remove the need for antibiotic selection often required to maintain plasmids. Such antibiotic pressure would not be available *in vivo* and hence the use of a plasmid may lead to the loss of the photonic signal during infection. Until recently, the most popular means of integrating reporter genes into the chromosome has been the use of transposons, such as the mini-*Tn*5 derivatives developed by Winson *et al.* (1998) for use in Gram-negative bacteria and the *Tn*4001 derivative developed by Francis *et al.* (2001) for use in Gram-positive bacteria. Using this strategy, transposon mutant pools are obtained and screened for derivatives with high expression of the reporter gene of interest, and which remain pathogenic (Francis *et al.*, 2001; Kuklin *et al.*, 2003; Park *et al.*, 2003; Wiles *et al.*, 2004; Kadurugamuwa *et al.*, 2005b; Rajashekara *et al.*, 2005;). While successful, this strategy can be extremely labour-intensive and is very reliant on the use of appropriate models to screen for attenuation. Furthermore, lack of attenuation in one model does not necessarily exclude a gene from playing a role in other models. Recently, Riedel *et al.* (2007a) constructed a novel vector (p16S*lux*), containing a temperature-sensitive Gram-positive origin of replication and a region of homology to the 16S rRNA gene. Using this construct, the authors were able to develop bioluminescent strains of *E. coli, Salmonella enterica, Enterobacter sakazakii, Shigella flexneri, Yersinia enterocolitica* and *Pseudomonas aeruginosa*. Furthermore, no differences were observed in bacterial load in the organs of mice infected with *C. rodentium, S. enterica* serovar Typhimurium and *P. aeruginosa* tagged using p16S*lux* and the wild-type strains (Riedel *et al.*, 2007a). Unfortunately, this strategy was unsuccessful in a number of Gram-positive bacterial species, suggesting that Gram-positive bacteria are more sensitive to the disruption of a copy of the 16S rRNA gene. Although not yet widely used, an alternative approach exploits bacteriophage integrase genes and attachment sites to direct the site-specific, single-copy integration of vectors into the bacterial chromosome. This system has the advantage of using previously characterized integration sites known not to affect bacterial virulence in defined models, and has been successfully used in *S. aureus, Listeria monocytogenes* and *M. tuberculosis* (Bron *et al.*, 2006; Steinhuber *et al.*, 2008; Andreu *et al.*, 2010;). Finally, another strategy being pursued involves mining the genome for apparently ‘null’ sites. Such sites may be identified by their apparent redundancy, a lack of homology to known virulence factors or by a lack of expression during *in vivo* microarray studies. However, it is important to note that such a strategy may have unexpected consequences. There are many genes with no ascribed function that may play a role during infection. Likewise, lack of expression in microarray studies does not necessarily exclude a gene from being involved in models other than the one in which the studies were undertaken.

### Selection of an appropriate reporter

The suitability of a given reporter gene for developing bioluminescent/fluorescent microorganisms will depend on numerous factors. If a fluorescent phenotype is desired, the FP will have to fulfil several criteria including suitable excitation and emission wavelengths, photostability, brightness, and maturation speed. It should be noted that for whole-body imaging, it is often not essential that the FP is available as a monomer; this is mostly imperative for cell biology applications where the FP is used as a tag for another protein. If oligomerization is tolerated by the host expressing the reporter gene, tandem dimers might be preferred because of often higher brightness.

If a luminescent phenotype is desired, the choice of luciferase system will largely depend on the microorganism of interest. A significant advantage of the bacterial luciferase system (*lux*) is the ability to express the enzymes for substrate synthesis, thus avoiding the need for exogenous addition of substrate. Despite this inherent advantage, the *lux* operon has remained almost exclusively used in bacteria, with almost all other microorganisms being labelled with genes encoding for eukaryotic luciferases (Table 6). The exception to this is the yeast *Saccharomyces cerevisiae*, which has been rendered bioluminescent using the *lux* operon (Gupta *et al.*, 2003). However, this required coexpression of the *frp* gene from *Vibrio harveyi*, encoding an NADPH-FMN oxidoreductase, to generate a detectable signal above background.

**Table 6:** Noninvasive imaging studies utilizing bioluminescent microorganisms

Microorganism	Reporter	Reference
Bacteria		
*Bacillus anthracis*	lux	Glomski *et al.* (2007a,b);, Sanz *et al.* (2008), Crawford *et al.* (2009), Loving *et al.* (2009)
*Bifidobacterium breve*	lux	Cronin *et al.* (2008)
*Brucella melitensis*	lux	Rajashekara *et al.* (2005, 2006), Radhakrishnan *et al.* (2009)
*Burkholderia pseudomallei*	lux	Owen *et al.* (2009)
*Citrobacter rodentium*	lux	Wiles *et al.* (2006b, 2007, 2009), Bishop *et al.* (2007), Gibson *et al.* (2008, 2010), Riedel *et al.* (2007a), Dennis *et al.* (2008), Hemrajani *et al.* (2008), Symonds *et al.* (2009)
*Edwardsiella ictaluri*	lux	Karsi *et al.* (2006)
*Escherichia coli*	lux, luc	Rocchetta *et al.* (2001), Jawhara & Mordon (2004, 2006), Demidova *et al.* (2005), Lane *et al.* (2007), Foucault *et al.* (2010)
*Haemophilus influenza*	lux	Mason *et al.* (2005), Novotny *et al.* (2005), Jurcisek *et al.* (2007), Bookwalter *et al.* (2008)
*Listeria monocytogenes*	lux	Hardy *et al.* (2004, 2006, 2009), Riedel *et al.* (2007b, 2009), Disson *et al.* (2008, 2009)
*Mycobacterium bovis* BCG	lux	Heuts *et al.* (2009)
*Mycobacterium smegmatis*	lux, luc	Andreu *et al.* (2010)
*Mycobacterium tuberculosis*	lux, luc	Andreu *et al.* (2010)
*Proteus mirabilis*	lux	Kadurugamuwa *et al.* (2005b), Burkatovskaya *et al.* (2006)
*Pseudomonas aeruginosa*	lux	BitMansour *et al.* (2002), Hamblin *et al.* (2003), Kadurugamuwa *et al.* (2003a,b);, Demidova *et al.* (2005), Burkatovskaya *et al.* (2006), Riedel *et al.* (2007a), Ramphal *et al.* (2008)
*Pseudomonas fluorescens*	lux	Sedgley *et al.* (2004, 2005)
*Salmonella enterica* Typhimurium	lux	Contag *et al.* (1995), Monack *et al.* (2004), Burns-Guydish *et al.* (2005, 2007), Riedel *et al.* (2007a)
*Salmonella enteritidis*	lux	Brovko *et al.* (2003)
*Staphylococcus aureus*	lux	Francis *et al.* (2000), Kuklin *et al.* (2003), Kadurugamuwa *et al.* (2003a, b, 2004), Gad *et al.* (2004), Wright *et al.* (2005), Xiong *et al.* (2005), Yu *et al.* (2005), Burkatovskaya *et al.* (2006), Mortin *et al.* (2007), Steinhuber *et al.* (2008), Engelsman *et al.* (2009)
*Staphylococcus epidermidis*	lux	Vuong *et al.* (2008)
*Streptococcus pneumonia*	lux	Francis *et al.* (2001), Echchannaoui *et al.* (2002), Malley *et al.* (2003), McCullers & Bartmess (2003), Orihuela *et al.* (2003, 2004), Kadurugamuwa *et al.* (2005a,c);, McCullers *et al.* (2007), Karlstrom *et al.* (2009), Kirby *et al.* (2009), Mook-Kanamori *et al.* (2009)
*Streptococcus pyogenes*	lux	Park *et al.* (2003)
Fungi		
*Aspergillus fumigatus*	luc	Brock *et al.* (2008)
*Candida albicans*	luc	Doyle *et al.* (2006a)
Parasites		
*Entomeaba histolytica*	luc	Asgharpour *et al.* (2005)
*Leishmania amazonensis*	luc	Lang *et al.* (2005)
*Leishmania major*	luc	Lecoeur *et al.* (2007, 2010)
*Plasmodium berghei*	luc	Franke-Fayard *et al.* (2005, 2008), Amante *et al.* (2007), Ploemen *et al.* (2009), Spaccapelo *et al.* (2010)
*Toxoplasma gondii*	luc	Hitziger *et al.* (2005), Saeij *et al.* (2005), Lambert *et al.* (2006), Boyle *et al.* (2007), Dellacasa-Lindberg *et al.* (2007), Vyas *et al.* (2007), Di Cristina *et al.* (2008)
*Trypanosoma brucei*	ruc	Claes *et al.* (2009), Vodnala *et al.* (2009)
*Trypanosoma cruzi*	luc	Hyland *et al.* (2008)
Viruses		
Equine encephalitis virus	luc	Gardner *et al.* (2008)
Herpes simplex virus Type I	luc, rluc	Luker *et al.* (2002, 2003), Krug *et al.* (2004), Luker & Leib (2005), Burgos *et al.* (2006)
Infectious hematopoietic necrosis virus	rluc	Harmache *et al.* (2006)
Koi herpes virus	luc	Costes *et al.* (2009)
Murine gammaherpes virus	luc	Hwang *et al.* (2008, 2009), Jia *et al.* (2010)
Murine herpes virus 4	luc	Gill *et al.* (2009), Milho *et al.* (2009)
Sendai virus	luc	Miyahira *et al.* (2009)
Sindbis virus	luc	Cook & Griffin (2003), Tseng *et al.* (2004)
Vaccinia virus	luc	Luker *et al.* (2005), Hung *et al.* (2007), Rivera *et al.* (2007)
Varicella zoster virus	luc	Zhang *et al.* (2007)

Of the *lux* operons cloned to date, that of *P. luminescens* is ideally suited for *in vivo* use as the luciferase has an optimum temperature range that lies within the body temperature of mammalian tissues (Szittner & Meighen, 1990). However, in general, it is not well-expressed by Gram-positive bacteria and modified versions are available in which the operon has been reorganized (*luxABCDE*) and Gram-positive ribosome-binding sites inserted (Francis *et al.*, 2000; Qazi *et al.*, 2001;). Furthermore, Craney *et al.* (2007) reported the construction of a synthetic *lux* operon, codon optimized for expression in high-GC bacteria such as *Streptomyces* sp.

It is important to note that bioluminescence from *lux*- and *luc*-expressing microorganisms is related to an organism's metabolic activity. This is due to the reliance of the luciferases on microbial metabolites, mainly FMNH_2_ and ATP, respectively. This is exemplified by the decrease in the luminescence that has been observed when many *lux*-expressing bacterial species enter stationary phase during *in vitro* growth (Francis *et al.*, 2001; Hardy *et al.*, 2004; Wiles *et al.*, 2004;). A similar finding has been reported for *lux*-expressing *Leishmania amazonensis* (Lang *et al.*, 2005) and *M. smegmatis* (Andreu *et al.*, 2010). Although this could represent a handicap for the study of, for example, dormant microorganisms *in vivo*, it can also be a powerful tool to gain insights into an organism's metabolic state during infection, as well as for the rapid detection of drugs that act on metabolism. In contrast, the bioluminescence of *Gluc*-expressing cells appears to be independent of cofactors that become limited during stationary phase (Wiles *et al.*, 2005b; Andreu *et al.*, 2010;).

### Maximizing detection limits

It is clear from the published literature that the *in vivo* limits of detection of labelled microorganisms vary considerably; this will be the result of a combination of numerous factors (Table 3). Glomski *et al.* (2007b) reported that the detection limits for *lux*^+^*B. anthracis* using the IVIS100 system (Xenogen, now part of Caliper Life Sciences, Alameda) were between 10^3^ and 10^5^ bacteria in different organs, and were a reflection of differences in organ location and pigmentation. Similarly, using the Xenogen IVIS50 system, Wiles *et al.* (2004) found the detection limits for *lux*^+^*C. rodentium* to be approximately 10^3^ bacteria within a foci. These limits are likely to improve as the sensitivity of the imaging systems improves.

However, as molecular microbiologists developing fluorescent/bioluminescent microorganisms, there are a number of factors that are somewhat within our control, namely: (1) levels of reporter gene expression, (2) wavelength of light emitted (as a result of the reporter gene chosen), (3) excitation wavelength (fluorescence), and perhaps, to some extent, (4) the availability of cofactors. The manner in which these factors may be manipulated to improve detection limits are discussed below. However, it is important to stress that the maintenance and expression of high levels of recombinant DNA may place an unwelcome metabolic burden on many microorganisms. Indeed, a number of researchers have described attenuation in *lux*- and GFP-expressing strains (Rocchetta *et al.*, 2001; Bumann, 2002; Hardy *et al.*, 2004; Sanz *et al.*, 2008;).

#### Reporter gene expression

In addition to copy number (discussed previously), robust levels of reporter gene expression are a function of transcriptional and translational signals. As a result, the selection of appropriate promoters is important for optimizing reporter levels. One area of optimization that is yet to be fully explored is that of codon usage. While this has proved unnecessary for most organisms expressing the *lux* operon, the sequence of the *lux* genes is AT-rich (>69%) and as a result they are not expressed efficiently in high-GC bacteria such as *Streptomyces coelicolor*. Furthermore, in *S. coelicolor* there is only one tRNA for the leucine-encoding codon (TTA), encoded by *bldA*, which is developmentally regulated in a number of *Streptomyces* spp. However, there are 63 TTA codons in the native *lux* operon. Craney *et al.* (2007) constructed an entirely synthetic *lux* operon lacking TTA codons and in which the majority of codons end in a G or C. The synthetic operon was found to be functional in *S. coelicolor* and to accurately report complex developmental gene expression patterns (Craney *et al.*, 2007). Furthermore, codon optimizing the firefly luciferase for *M. tuberculosis* resulted in a 30-fold increase in signal (Andreu *et al.*, 2010). Perhaps most impressive of all, a combination of codon optimization, removal of cryptic splice sites and retroviral modification was used to engineer an enhanced firefly luciferase vector that allowed the generation of highly bioluminescent T cells, permitting the detection of as few as three cells implanted subcutaneously into mice (Rabinovich *et al.*, 2008). However, it is important to note that codon optimization may have unforeseen effects; the *lux* operon codon optimized for *M. tuberculosis* was found to be nonfunctional (Andreu *et al.*, 2010). While we have not explored the basis for this inactivity, there is a suggestion from the optimized sequence that after transcription, the DNA may form secondary structures that impede translation.

#### Wavelength of light emitted

For bioluminescence, it is possible that utilizing the firefly luciferase rather than the *lux* operon may result in improvements in the limits of detection *in vivo*. This would almost certainly be due to the red-shifted emission spectra (Zhao *et al.*, 2005a) and increased efficiency of the firefly luciferase (Ando *et al.*, 2007). However, unlike the *lux* operon, the generation of a bioluminescent signal is entirely dependent on the efficient delivery of exogenous substrate.

#### Increasing cofactor availability

For bacteria expressing the *lux* operon, it is possible that the availability of aldehyde and FMNH_2_ are limiting factors. Indeed, cloning an extra promoter in front of *luxCDE* to boost substrate synthesis resulted in a sixfold higher signal in *M. smegmatis* (Andreu *et al.*, 2010) and *S. aureus* (Mesak *et al.*, 2009). Furthermore, in yeast, coexpression of *luxAB* together with the *frp* gene, encoding an NADPH–FMN oxidoreductase from *V. harveyi*, led to a 100-fold increase in luminescence (Gupta *et al.*, 2003). Similarly, Mesak *et al.* (2009) cloned the *V. harveyi frp* into their constructs for use in *S. aureus* but were unable to obtain any transformants with these plasmids. The authors suggested that the apparent toxicity of *frp* to *S. aureus* may be due to the generation of superoxide (O_2_^−^) as a result of the auto-oxidation of flavoproteins.

## Applications of BPI to infectious disease research

The use of BPI for studying infectious microorganisms *in vivo* represents an elegant and simple approach, avoiding many of the problems associated with conventional methods, such as those based on obtaining viable counts. The most widespread conventional approach involves euthanizing groups of animals at numerous time points, followed by the preparation of suspensions of organs/tissues of interest, which are then plated onto selective media and the number of viable microorganisms determined. Although providing a picture of the progression of the infection, this cumbersome technique has many limitations. Firstly, such data provide researchers with snapshots of the infection only at the particular time points and in those organs/tissues analysed. Importantly, it is impossible to discern what happened between any two given time points, or whether the infection progressed beyond the organs/tissues selected for analysis. Secondly, the dynamics of the infection cannot be followed in the same group of animals; the data at each time point is obtained using different animals, which may lead to the introduction of confounding errors. Thirdly, the results are retrospective, thus preventing the ability to make ‘on-the-spot’ decisions based on the level of infection, such as whether to start a treatment. In contrast, BPI offers the ability to gather real-time information on infection progression using the same group of animals. Moreover, because the whole animal is imaged, microorganisms can be detected at any location including those previously unknown, provided they are present in sufficient numbers to be detected above background. BPI therefore maximizes the amount of information that can be obtained from a single experiment while drastically reducing the number of animals required. The use of BPI can result in important new insights into the niches exploited by pathogens during infection, challenging conventional dogma and opening new avenues for research into therapeutic agents and vaccines.

## Noninvasive imaging of bioluminescence in infectious disease research

BPI using bioluminescence is now well-established in the field of infectious disease research and has utilized a number of approaches: (1) monitoring the numbers and location of microbial pathogens, (2) monitoring the timing and location of microbial gene expression (e.g. those genes involved in virulence), (3) monitoring the efficacy of antibiotic treatment or vaccination, and (4) monitoring the host response.

### Monitoring pathogen numbers and location: investigating the dynamics of infection in real-time

The first reported use of BPI to track pathogenic microorganisms in animals was presented by Contag and colleagues 15 years ago using *S. enterica* serovar Typhimurium transformed with the *luxCDABE* operon (Contag *et al.*, 1995). This seminal publication not only proved that bacteria could be detected in intact living mice, but that the technique could be used to impart important information regarding the infection process. Indeed, the authors used BPI to illustrate differences in the colonization pattern of three *Salmonella* strains with varying degrees of virulence, as well as the effects of inoculation route and mouse strain used. In the intervening years, the validity of BPI in outlining the dynamics of growth and dissemination that encompass infection *in vivo* has been proven for a number of bioluminescently labelled microbial pathogens, including bacteria, viruses, fungi and parasites (Table 6).

#### Bacterial pathogens

*Bacillus anthracis* is a sporulating Gram-positive bacterium responsible for causing anthrax. Anthrax can progress in three different forms depending on the route of infection: cutaneous, gastrointestinal and inhalational. Using spores of *B. anthracis* expressing the *lux* operon, Glomski *et al.* (2007b) sought to investigate the patterns of growth associated with the three different types of infection. Because spores are metabolically inert, germination could be easily determined by the appearance of the luminescent signal. The researchers found that germination and initial growth take place at the site of inoculation in both cutaneous and inhalational infections, ruling out the previously thought essential involvement of the draining lymph nodes. If this holds true for human infection, this finding has important implications for the time within which antibiotic therapy should be administered. Furthermore, image-guided dissection allowed the authors to point to the Peyer's patches (aggregations of lymphoid tissue in the walls of the intestines) as the primary site of bacterial growth after intragastric inoculation. All routes of infection progressed to the draining lymph nodes, spleen and eventually lungs and blood. Luminescence correlated with bacterial counts in the ear, superficial parotid lymph node, spleen and right lung. However, because of differences in organ location and pigmentation, the limits of detection ranged from 3 × 10^3^ CFU for the ear to 2 × 10^5^ CFU in the spleen. Likewise, quenching of the light emitted from the left lung by the heart was detected. The study utilized a capsulated nontoxinogenic strain of *B. anthracis;* however, for safety reasons toxinogenic noncapsulated strains are frequently used in combination with sensitive mouse strains. To assess the consequences of the lack of capsule in the growth and dissemination of *B. anthracis*, a luminescent *lux*^*+*^ toxinogenic noncapsulated strain was used to compare the infection dynamics with that of the previously described capsulated nontoxinogenic strain in a cutaneous model of infection (Glomski *et al.*, 2007a). Importantly, the researchers found that the noncapsulated toxinogenic bacteria were confined to the site of inoculation for longer than the capsulated nontoxinogenic strain. Furthermore, after progressing to the draining lymph nodes, dissemination to other organs was also delayed and, in contrast to the capsulated nontoxinogenic strain, colonization of the spleen was minimal. The use of BPI has therefore allowed researchers to gain insights into the patterns of growth and dissemination of *B. anthracis*, describing new sites of infection and identifying previously unknown colonization differences between strains. Importantly, these findings highlight the need for a careful choice of infection model according to the experimental question being addressed.

*Listeria monocytogenes* is another example in which the use of BPI has uncovered novel niches during infection. While using a *lux*^*+*^*L. monocytogenes* strain, Hardy *et al.* (2004) discovered a strong focal signal in the thoracic region of most of the mice that had been either intravenously or orally inoculated. Image-guided dissection allowed the researchers to identify the gallbladder as the source of the signal. Moreover, histological analysis showed *L. monocytogenes* growing extracellularly in the lumen of the gallbladder, a very inhospitable environment where only *S. enterica* serovar Typhi was previously thought to grow. In fact, the gallbladder is where *Salmonella* is located in asymptomatic carriers who excrete the pathogen within the faeces, thus providing an efficient means of transmission (Levine *et al.*, 1982). Similarly, the gallbladder could constitute the niche by which *L. monocytogenes* is transmitted by asymptomatic carriers. To address this question, Hardy *et al.* (2006) monitored bioluminescent *L. monocytogenes* growing in the gallbladder after inducing its contraction either by feeding mice with milk or by injecting them with the hormone cholecystokinin. To succeed it was very important to ensure that the bacteria were growing in the gallbladder at the point of inducing contraction. This would have been quite difficult using retrospective CFU assays but was easily achieved using BPI. Using a *lux*^*+*^ attenuated *L. monocytogenes* strain, which could be administered as a larger dose, the researchers reported seeing the photonic signal leaving the gallbladder and moving along the gut until it reached the point where faeces were being formed. The signal appeared and disappeared as it travelled through the gut, which was related to changes in the location of the bacteria relative to the surface of the mice as proved by *ex vivo* imaging. This is a good example of how the signal intensity depends not only on the amount of bacteria but also on their location. *Listeria monocytogenes* was also detected in the faecal pellets, indicating that faeces may represent a source for reinfection or new infections. More recently, BPI has been used to study bone marrow infection by *L. monocytogenes* (Hardy *et al.*, 2009). As previously observed, infection of mice by this bacterium was cleared during the first hours, followed by dynamic relapses in different locations, including the bones. This complex pattern of infection would have been very difficult to identify using conventional methods because of the variety of sites of infection, the fluctuations in the levels of bacteria over time and inter-mouse variation. Luminescent signals in the bones were mainly located in the tibia and phalanges of the hind legs and lasted for several days particularly in the case of attenuated *lux*^*+*^*L. monocytogenes* strains. Image-guided dissection and histology showed that bacteria replicated in the bone marrow without causing any observable pathology. Importantly, the presence of bacteria in the bones was without any evident clinical symptoms, thus emphasizing the crucial role of BPI in the detection of this infection.

Brucellosis, caused by *Brucella melitensis*, is a disease that can progress as an acute or chronic infection affecting a broad range of tissues; hence it represents another case in which the use of BPI has the potential to make important contributions to our understanding of the infection. Rajashekara *et al.* (2005) analysed an acute infection model in susceptible and resistant mice using a *lux*^*+*^ strain. A similar pattern of dissemination was revealed in both mouse strains with signals detected at the site of inoculation (peritoneum), in the inguinal lymph nodes, liver, and spleen, and in two newly described locations of relevance for human infection: the testes and submandibular regions. In addition, a chronic infection model was developed in resistant mice and in susceptible mice infected with a low dose, which was characterized by waves of growth and clearance of *B. melitensis* in the submandibular region and tail. The *in vivo* imaging results correlated well with *ex vivo* imaging and CFU counts for all the organs tested.

Meningococcal sepsis is another infectious disease whose research has also benefitted from the application of BPI. Sjolinder & Jonsson (2007) used a *lux*^*+*^*Neisseria meningitidis* strain to infect CD46 transgenic mice, which are susceptible to meningococcal disease in contrast to resistant wild-type mice. The infection resulted in three distinct disease patterns: sepsis, meningitis and mild disease. Sepsis and meningitis were found to result in death within 3 days, whereas mild disease was characterized by clearance followed by relapses with bacteria either localized in the central nervous system or the circulation resulting in bacteraemia. Additionally, strong photonic signals were detected in the thyroid gland and nasal region. Further analysis of the thyroid gland indicated that colonization was related to lower levels of the thyroid hormone in the transgenic mice, which would point at an impaired thyroid function as a risk factor for meningococcal disease. CFU and immunohistochemical analysis of the nasopharynx gave the first evidence *N. meningitidis* translocates from blood to the respiratory mucosa. Moreover, using a bioluminescent *N. meningitidis* strain deficient in the pilus-associated adhesin PilC1, the researchers found that this protein plays a crucial role in this process.

Another area of research in which use of BPI represents an important improvement is in the study of biofilm formation *in vivo*. Conventional methods require removing the device that acts as support for the biofilm followed by detaching and quantification of the bacteria, and therefore do not allow longitudinal monitoring of the infection. The usefulness of BPI in this field was first demonstrated by Kadurugamuwa *et al.* (2003a) using implanted catheters colonized by either *lux*^*+*^*S. aureus* or *lux*^*+*^*P. aeruginosa*. In both cases, bioluminescence correlated to CFU numbers throughout the experiment. More recently, BPI was used to monitor *S. aureus* in a soft tissue implant infection model (Engelsman *et al.*, 2009). In contrast to other biofilm studies, in this model growth of the bacteria was not restricted to the device; instead, an infected mesh was subcutaneously implanted, thus allowing the bacteria to spread into the surrounding tissues. Bioluminescence over the mesh area correlated with *ex vivo* bacteria enumeration by confocal microscopy.

Recently the dynamics of faecal–oral transmission was studied using a *lux*^+^ derivative of *C. rodentium*, a natural murine pathogen (Wiles *et al.*, 2005a). In this model, a single mouse, infected by oral gavage with bacteria grown in culture, is reintroduced into a cage containing its littermates and allowed to infect them. BPI was used to study the tissue distribution of bacteria in animals infected by the two routes and revealed startling differences. In particular, bacteria grown in culture were found to require a large infecting dose and initially small numbers colonize the caecum for a period of several days before going on to infect the lower reaches of the colon (Wiles *et al.*, 2004, 2006b). By contrast, *C. rodentium* shed in infected murine stool was shown to have an approximately 1000-fold lower infectious dose, indicating that after adaptation to the murine host *C. rodentium* becomes relatively hyperinfectious when subsequently shed into the environment (Wiles *et al.*, 2005a), and remain in this state for several days (Bishop *et al.*, 2007). Furthermore, host-passaged bacteria were found to immediately colonize the colon, suggesting that strains grown in culture require a period in which to adapt to the new environment. The classic definition of refinement described earlier can be extended beyond minimizing pain and suffering to ensuring the model used is the best representation of the disease under study. In the case of *C. rodentium*, a model for human gastrointestinal pathogenic *E. coli*, the insights provided by BPI in comparing infections initiated by oral gavage and natural transmission resulted in the development of an animal model that more realistically models the human infection, while requiring far fewer animals to undergo the invasive oral gavage procedure.

#### Fungal pathogens

To date, the infection dynamics of two fungal species have been investigated using BPI, *Aspergillus fumigatus* and *Candida albicans. Aspergillus fumigatus* causes life-threatening aspergillosis in immunocompromised patients while *C. albicans* is an opportunistic pathogen that commonly resides in the human digestive system and vaginal tract as a commensal yeast. Brock *et al.* (2008) developed bioluminescent *A. fumigatus* using a derivative of the firefly luciferase gene, codon optimized for use in mammalian cells, and found that differences in the light emission of the transformants was related to the number of copies of luciferase introduced. The bright transformant C3, found to have four luciferase copies, was selected for further study. Using a model of invasive pulmonary aspergillosis in which corticosteroid-treated mice are intranasally inoculated with high numbers of conidia, the researchers determined that *A. fumigatus* C3 was not attenuated, leading to respiratory failure 3–4 days after infection. Interestingly, light emission was found not to be a good indicator of fungal load in this model; the photonic signal from the lungs peaked 1 day postinfection and then decreased while the clinical symptoms of infection continued indicating a progression of infection. Furthermore, high fungal densities were evident by histology. The authors suggested that the disparity between light emission and fungal load may be due to inefficiencies in luciferin distribution as a result of the severe clinical symptoms exhibited, as well as the presence of pulmonary lesions that may have been severe enough to restrict oxygen dispersion in the bronchoalveolar tree. Indeed, the researchers found a strong increase in light emission when luciferin was directly administered to lungs *ex vivo*.

Doyle *et al.* (2006b) developed bioluminescent *C. albicans* using a codon optimized derivative of the firefly luciferase gene. The researchers found that while luciferase activity in protein extracts taken from *C. albicans* growing as yeast or hyphae were almost identical, light output from the hyphal stage was massively reduced (Doyle *et al.*, 2006a). This was attributed to a reduced ability of luciferin to cross the hyphal cell wall and was a disappointing finding as in *C. albicans* hyphal growth is associated with the fungus in its pathogenic, infectious form. Indeed, Doyle and colleagues found that the bioluminescent signal was generally too low to monitor chronic systemic infection *in vivo* using BPI. In contrast, BPI was successful in monitoring *C. albicans* infection in a vulvo-vaginal model as this infection is characterized by the presence of both the hyphal and yeast morphologies, with the sloughing off of the latter cell type.

#### Parasites

*Trypanosoma* spp. are eukaryotic parasites endemic in Africa, Latin America and Asia. *Trypanosoma cruzi* is the causative agent of Chagas disease; acute disease is typically characterized by high parasitism, fever and lymphadenopathy, which commonly progresses to a chronic phase where cardiac alterations or gastrointestinal disorders are observed. Hyland *et al.* (2008) developed firefly luciferase-expressing *T. cruzi* and were able to follow parasite dissemination over the course of a 25-day infection using BPI. *Trypanosoma brucei* subspecies *brucei* is transmitted by the tsetse fly and causes human African trypanosomiasis, also known as sleeping sickness. Claes *et al.* (2009) generated *T.b. brucei* stably expressing the *Renilla* luciferase and characterized parasite infection dynamics in intraperitoneally infected mice. Interestingly, the authors found an abundance of parasites in the testes of infected male mice, but no apparent tropism for the sexual organs of female mice. Furthermore, *T.b. brucei* could be observed extravascularly in the testes but not in the seminiferous duct, suggesting sexual transmission was unlikely. Indeed, no female mice became infected when mated with *T.b. brucei*-infected males. Furthermore, no offspring were generated, suggesting that the presence of parasites in the testes leads to male sterility. It would be interesting to determine whether the tropism of *T.b. brucei* for the testis holds true in models that more realistically mimic the natural route of parasite transmission, for example subcutaneous injection to mimic delivery from the tsetse fly. Importantly, the researchers noted that administration of the coelenterazine substrate required for generation of a photonic signal resulted in nonhomogenous distribution with different patterns of light emission observed after intraperitoneal and intravenous delivery.

*Leishmania* spp. are intracellular protozoan parasites transmitted from the sandfly during a blood meal to a variety of mammalian hosts. These dimorphic parasites exist as extracellular flagellated promastigotes in the insect vector and as obligate intracellular amastigotes in the mammalian host. Lang *et al.* (2005) generated *Leishmania amazonensis* recombinants stably expressing the firefly luciferase gene and visualized the presence of metacyclic promastigotes into the ear dermis of mice using BPI. Bioluminescent signals, measured at the inoculation site and in the draining lymph node, correlated well with classical methods for quantification of parasites and allowed the monitoring of parasite loads before any clinical signs of leishmaniasis were detectable. Similarly, Lecoeur *et al.* (2007) developed firefly luciferase-expressing *Leishmania major* and determined the limits of detection to be approximately 5000 parasites within the murine ear.

*Toxoplasma gondii* is an obligate intracellular parasite that can cause severe disease in individuals with immature or suppressed immune systems. The parasite has two forms that express distinct surface antigens; the rapidly dividing or tachyzoite form is associated with acute infection, while the bradyzoite form is associated with asymptomatic chronic infection. A number of researchers have reported the development of firefly-expressing derivatives of different *T. gondii* strains (Hitziger *et al.*, 2005; Saeij *et al.*, 2005; Lambert *et al.*, 2006; Vyas *et al.*, 2007; Di Cristina *et al.*, 2008;). Interestingly, Saeij *et al.* (2005) reported that all mice infected with *T. gondii* strain S23-luc7 that died in the acute phase of infection developed a signal around the ventral side of the neck, which was identified as originating from the cervical lymph nodes and is similar to the situation in humans. However, dissection of animals also showed a clear signal emanating from specific regions of the brain, which was not observed when the animals were imaged while anaesthestized. In contrast, Hitziger *et al.* (2005) were able to visualize the dissemination of *T. gondii* strain RH to the brain, eyes and testes in addition to the spleen and liver. Such differences could be due to strain differences as well as imaging parameters; importantly, visualizing the signal emanating from the eyes and brain required the bodies of the animals to be covered with black card to block the stronger photon emission of *T. gondii* in other niches.

Firefly luciferase-expressing malaria parasites have been used in a number of studies to monitor the interactions between *Plasmodium berghei* and its murine host. Initial studies concentrated on investigating the blood-borne stages of infection. For example, *Plasmodium*-infected red blood cells are known to adhere to the endothelial cells of the microvasculature of numerous deep tissues. This process, known as sequestration, has long been considered to lead to the pathology of cerebral malaria. However, using BPI, Franke-Fayard *et al.* (2005) determined that sequestration was not the basis for murine cerebral malaria and revealed an unexpected tissue (adipose) in which sequestration occurs. These studies relied on establishing malaria in mice by administering *Plasmodium*-infected red blood cells by the intraperitoneal route. Importantly, the liver is the main site for intracellular development of *Plasmodium* sporozoites in humans and rodents upon being bitten by an infected mosquito. Ploemen *et al.* (2009) utilized firefly luciferase-expressing *P. berghei* to study this important phase of the parasites life cycle. Parasites were administered to mice either by intravenous injection of sporozoites or by mosquito bite, and the liver stage of *Plasmodium* was visualized by BPI. In this manner, the authors were able to discriminate less than five infected hepatocytes per liver using planar luminescence imaging. Furthermore, this study was one of the few reports in the literature of bioluminescence tomographic imaging utilizing the now discontinued IVIS 3D system, which allowed the discrimination of individual infected hepatocyctes.

#### Viral pathogens

The first report of BPI to monitor viral pathogens was by Luker *et al.* (2002), who utilized a dual firefly and *Renilla* luciferase-expressing derivative of herpes simplex virus type 1 (HSV-1). While viral infection in mouse footpads, peritoneal cavity, brain, and eyes could be detected after administration of luciferin, the activity of *Renilla* luciferase could only be visualized after direct administration of coelenterazine to infected eyes and not following the systemic delivery of substrate. Importantly, the photonic signal derived from the expression of firefly luciferase *in vivo* correlated directly with input titers of recombinant virus used for infection. One minor drawback of utilizing BPI to study microbial pathogenesis is that the technique requires bioluminescent derivatives of different strains to be developed. This may result in unforeseen effects on virulence, in addition to the time and resources required. Luker & Luker (2010) developed a novel alternative approach to this strategy with the construction of a transgenic mouse that expresses firefly luciferase only in response to HSV-1 infection (Luker *et al.*, 2006). After infection with three different strains of HSV-1, luciferase expression in the transgenic mice reproduced the established spatial and temporal progression of infection, with the photonic signal reflecting the input viral titers. However the authors noted that the lower limits of detection were approximately 10-fold higher than when using luciferase-expressing HSV-1. Importantly, this decrease in sensitivity resulted in a delay of 1–2 days before viral infection could be detected. Furthermore, relatively high basal levels of luciferase expression were observed in the paws, ears and tail, limiting the usefulness of the reporter mouse strategy for studies utilizing the footpad inoculation route.

Sindbis virus (SV) is an alphavirus that causes encephalitis and paralysis in mice and therefore serves as an excellent model for studying acute viral encephalitis. For alphaviruses (and others found in plasma), viral spread from the periphery to the central nervous system has been difficult to study due to the confounding effects of free virus in the blood. Indeed, determination of replication in specific tissues requires the identification of infected cells by methods such as immunohistochemistry. As firefly luciferase requires ATP to generate light, Cook & Griffin (2003) developed firefly luciferase-expressing variants of SV and utilized BPI to visualize only virus actively replicating within cells. Mice were subcutaneously inoculated in the right rear foot and viral replication in the brain was always found to be preceded by replication in either the nose or lower spinal cord. This suggests that virus entry into the nervous system can occur by retrograde axonal transport either from neurons innervating the initial site of replication or from the olfactory epithelium after viraemic spread. BPI was also utilized to examine the reactivation of gammaherpesviruses, which can establish life-long persistency inside host cells. Using a firefly luciferase-expressing derivative of murine gammaherpesvirus 68 (MHV-68), Hwang *et al.* (2008) visualized the spontaneous reactivation of MHV-68 from latency, as well as following treatment with either a proteasome inhibitor or an immunosuppressant agent. Furthermore, BPI revealed viral replication in the salivary glands, an important finding that suggests that MHV-68 may also be transmitted through saliva, similar to a number of human herpesviruses.

#### Host range of BPI

The majority of reports of BPI to date involve using mice as the host species. The reasons for this are twofold: (1) mice account for a majority of the animals used in scientific procedures (Trull & Rich, 1999; UKHO, 2009;), and (2) their small size minimizes attenuation of the photonic signal caused by the distance the signal must travel to reach the surface. However, the technique is not limited to mice and has been applied to other species, most notably to study gene transfer efficiency in infant monkeys (the long-tailed macaque, *Macaca fascicularis*) (Tarantal *et al.*, 2006). In infectious disease research, BPI has been applied to a chinchilla model of *Haemophilus influenza* otitis media, allowing the noninvasive monitoring of the infection within the pharynx, eustachian tube, and middle ear after intranasal and transbullar inoculation (Novotny *et al.*, 2005). The limit of detection for BPI in the nasopharynx was found to be 10^5^ CFU mL^−1^ of nasopharyngeal lavage, less than the conventional CFU method. In contrast, detection of luminescence within the middle ear cavity appeared to be more sensitive than culture, which could be related to the presence of a subpopulation of adherent cells that would be recovered by lavage less efficiently. Likewise, BPI was used to detect fetoplacental transmission of *L. monocytogenes* in a gerbil infection model (Disson *et al.*, 2008), and to monitor spatiotemporal progression of bacterial peritonitis in rats (Sharma *et al.*, 2010).

In addition to rodents, BPI has also been applied to the study of infection in fish. A *lux*^*+*^*Edwardsiella ictaluri* strain was used to study bacterial dissemination in channel catfish (Karsi *et al.*, 2006). Intraperitoneal inoculation was used to determine the limit of detection *in vivo*, which was found to be approximately 10^4^ CFU in non-albino fish. In addition, fish immersed in water containing the luminescent bacteria allowed the visualization of bacterial attachment to specific novel sites on the fish's surface. This was followed by spread of the bacteria to various areas and eventual dissemination through the whole body. Moreover, *ex vivo* analysis corroborated previously known target organs of infection (the kidneys, spleen and gills) while identifying new ones (e.g. the heart). Interestingly, the authors found that *E. ictaluri* infection was characterized by an initial period of stable bacterial numbers followed by a period of rapid bacterial replication and dissemination, suggesting that the organism must first reach a certain population density before causing disease. Once the bioluminescent signal reached approximately 10^8^–10^9^ photons s^−1^ cm^−2^ steridian (sr)^−1^, death of the host was imminent. In addition, Harmache *et al.* (2006) utilized BPI to investigate the spread of a novirhabdovirus, infectious hematopoietic necrosis virus (IHNV), in live rainbow trout. Expression of the *Renilla* luciferase in IHNV was found to have no effect on virulence and enabled viral dissemination to be visualized in numerous tissues including the oral cavity, esophagus/cardiac stomach region, kidney and spleen after addition of substrate into the water tank. Interestingly, the fin bases were found to be the portal of entry in fish exposed to IHNV by bath immersion. Similarly, Costes *et al.* (2009) determined the skin, and not the gills, to be the major portal of entry for Koi herpesvirus in carp using BPI.

### Studying virulence factors and gene expression *in vivo*

BPI can be used to gain insights into the *in vivo* role of microbial gene products, by comparing the infection dynamics of wild-type and mutant tagged strains. Moreover, the spatiotemporal expression of a particular gene of interest can be monitored by expressing reporter genes under control of the promoter of a given gene, provided expression is strong enough to generate a detectable signal. Rajashekara *et al.* (2005) first applied BPI to the study of virulence factors while screening a transposon library for a highly bioluminescent strain of *B. melitensis*. In addition to attenuation, revealed by reduced lethality, BPI allowed the researchers to easily identify differences in bacterial growth and dissemination between some of the mutant strains. More recently this approach has been used to study the role of a TIR domain-containing protein (TcpB) of *B. melitensis* during infection in mice (Radhakrishnan *et al.*, 2009). *In vitro*, the authors demonstrated that this protein plays a similar role to that of the Toll-like receptor adaptor protein TIRAP. Furthermore, using BPI, TcpB was found to be required for the initial growth and spread of *B. melitensis in vivo*. Similarly, this technique has been used to characterize the dissemination patterns of various *B. anthracis* mutant strains deficient in one or more toxin components (Loving *et al.*, 2009). In this manner, it has been found that while toxins are not required for germination or initial replication *in vivo*, the lethal toxin and, to a lesser extent, the oedema toxin are required for dissemination beyond the draining lymph nodes. In a similar work, BPI was used to monitor six *Streptococcus pneumoniae* mutant strains lacking previously recognized virulence factors (Orihuela *et al.*, 2004). Different inoculation routes were used to distinguish between defects in dissemination and inability to grow in certain tissues and, thereby to allocate the contribution of each virulence factor to disease progression. In yet another excellent example, BPI was used to study the involvement of the bacterial invasion proteins InlA and InlB in maternofetal transmission of *L. monocytogenes* (Disson *et al.*, 2008). A gerbil model was used because of the presence in this animal of functional receptors for both invasiveness proteins. Fetoplacental infection could be readily visualized using a bioluminescent wild-type strain either after oral or intravenous inoculation, whereas no signal in the foetus was detected when using a double-mutant strain under the same conditions. These results were correlated to foetal lethality and helped to prove the involvement of InlA and InlB in fetoplacental listeriosis *in vivo*.

Varicella-zoster virus (VZV) is a human alphaherpesvirus that causes varicella (chickenpox). VZV establishes lifelong latency in the host, with reactivation resulting in herpes zoster (shingles). VZV possesses a DNA genome of 125 kb that bears 70 unique ORFs; <20% of the VZV genome has been functionally characterized. VZV infection is restricted to human cells, and mouse models for VZV studies are limited to those that engraft human tissues in immunodeficient mice. Zhang *et al.* (2007) developed a firefly luciferase-expressing VZV derivative in which bioluminescence correlated with viral titers both *in vitro* and in infected thymus–liver implants in SCID-hu mice. Visualization of the kinetics of VZVLuc spread in mice using BPI demonstrated a general trend of initial exponential growth followed by a plateau once the viral infection within the limited implants had reached saturation. Furthermore, mutant viruses in which ORF1 and ORF2 were deleted demonstrated no discernible growth defect in thymus–liver implants, while virus lacking ORF3 grew slightly, but not significantly, slower than the wild-type virus. In contrast, the implants inoculated with virus lacking ORF0 produced photonic signals that were approximately 100-fold less than those of VZVLuc. These data suggest that VZV ORF1, ORF2 and ORF3 are dispensable, while ORF0 is required for optimal viral growth. Similarly, Gill *et al.* (2009) utilized a firefly luciferase-expressing variant of the murine gammaherpesvirus Murid herpesvirus-4 (MuHV-4) to investigate the relationship between viral thymidine kinase (TK), a gene classically essential for lytic replication in terminally differentiated cells, and host entry route. The authors found that while MuHV-4 lacking TK delivered to the lung or peritoneum were attenuated they were able to disseminate to lymphoid tissue. In contrast, MuHV-4 lacking TK delivered to the upper respiratory tract resulted in no detectable infection, suggesting TK, and by implication lytic replication, is required for MuHV-4 to establish a significant infection by a noninvasive route.

In spite of the extensive use of luciferases as reporters to study microbial gene expression *in vitro*, to our knowledge, carrying out such studies noninvasively in animals was not reported until 2005 with the publication of expression of the *sap* operon in a chinchilla model of *H. influenza* otitis media (Mason *et al.*, 2005). This operon has been implicated in resistance to antimicrobial peptides (Harris *et al.*, 2004), some of which are known to be important in innate immunity of the upper airway mucosa. Expression of *sapA* had been previously shown to be increased in *H. influenzae* recovered from the middle ear in the chinchilla model (Mason *et al.*, 2003). By fusing the *sap* promoter to the *lux* operon, transient expression could be detected in the middle ear, the eustachian tube and the pharynx. Bioluminescence was measured *in vivo* and CFUs were calculated in ear fluids and nasal lavages to work out the amount of bioluminescence per bacterial cell and therefore estimate variations in expression during infection.

Almost simultaneously, Wright *et al.* (2005) used BPI to study the expression and regulation of the *agr* system of *S. aureus* in a murine subcutaneous abscess model. The *agr* system, which stands for *accessory gene regulator*, is a quorum-sensing system that controls the expression of *S. aureus* virulence genes in response to autoinducing peptides (AIP). There are different *agr* specificity groups that produce specific AIP that competitively inhibit heterologous *agr* activation and therefore virulence. To better understand this process, Wright and colleagues constructed two bioluminescent *S. aureus* strains in which the *lux* operon was under the control of either an *agr* promoter or the β-lactamase promoter (for constitutive expression). They proved that both bioluminescence and abscess formation in the *agr* reporter strain specifically correlated with the presence of a cognate AIP whereas it could be inhibited by either heterologous AIP or a synthetic antagonist. Interestingly, an eclipse period with background levels of luminescence was detected between 8 and 24 h postinfection for both reporter strains, despite high bacterial loads. This effect was reversed when infection was carried out in mice depleted of polymorphonuclear leukocytes, the main immune responders to the infection. The authors concluded that the reduction in bioluminescence observed was likely due to a metabolic shutdown in the bacteria, possibly after phagocytosis by the immune cells. This highlights the importance of concurrently using a constitutive promoter as a control.

Steinhuber *et al.* (2008) reported no such problems in their studies of the expression of the *S. aureus* pore-forming alphatoxin Hla in implanted tissue cages. In this model, bioluminescence detected in mice infected with the constitutive reporter strains correlated with bacterial numbers throughout the length of the experiment. Conversely, expression of *hla* was not detected until 2 days postinfection, after which time it steadily increased until the end of the study. Moreover, the luminescent signal was higher when *lux* was expressed under control of the *hla* promoter in a strain deficient in SigB, known to repress *hla*, whereas no signal was detected in a strain lacking Sae, an activator of *hla*. Significantly, high bacterial densities were required to generate a detectable signal illustrating the importance of promoter strength.

Promoter strength also posed a problem for Sanz *et al.* (2008) who used *B. anthracis* strains in which the *lux* operon was expressed either under the control of the *sspB* spore promoter or the PA vegetative promoter to unravel the actual site of germination of this bacterium. The two reporters clearly enabled the researchers to differentiate between germination and vegetative growth *in vitro*. Furthermore, germination could be detected *in vivo* as early as 20 min after subcutaneous inoculation. However, the limits of detection were found to be much lower in the lungs where luminescence was not detected until 18 h postinfection even after administration of a high infectious dose. This delay in signal detection was due to attenuation of the light by the tissues as demonstrated by *ex vivo* imaging of the lungs. Despite this limitation, BPI allowed the researchers to determine the time and location of germination, directing further histological analysis. However, the luminescent strains were found to germinate at a slower rate and had a higher LD_50_ (dose at which 50% of the animals die) after subcutaneous and intranasal inoculation. Attenuation of *lux*^*+*^ strains has been noted previously for other bacteria such as *E. coli* (Rocchetta *et al.*, 2001) and *L. monocytogenes* (Hardy *et al.*, 2004), and has been attributed to the metabolic burden caused by maintenance and expression of the recombinant DNA.

Finally, the work of Lane *et al.* (2007) on uropathogenic *E. coli* constitutes one of the best examples of the use of BPI to visualize infection dynamics and gene expression *in vivo*. Using an *E. coli* strain in which the *lux* operon was constitutively expressed, the researchers could track dissemination of the infection from the bladder to the kidneys. Moreover, doing the same experiment, but using the flagellin promoter to drive expression of *lux*, Lane and colleagues could indirectly visualize flagella expression during infection, thus proving their involvement during ascending urinary tract infection. The authors observed a trail of bioluminescence from the bladder to the kidneys starting at 5 h postinfection, followed by increasing levels of signal in the kidneys and, contrary to the constitutive promoter, decreasing amounts of light in the bladder. Bioluminescence levels correlated with motility *in vitro* as well as with flagellin expression assessed by quantitative PCR both *in vitro* and in the bladder *in vivo*, thus proving the validity of this approach.

### Monitoring antibiotic treatment and vaccine efficacy

One of the main bottlenecks in vaccine and drug development is *in vivo* testing in animal models. This is primarily due to the fact that conventional viable count methods are laborious, time-consuming, and involve a huge number of animals, thus limiting its application to high-throughput screening. It soon became apparent that BPI could easily overcome this handicap and assessment of the usefulness of this method for antibiotic susceptibility testing constitutes a common theme in many of the publications in the field. In their seminal work, Contag *et al.* (1995) used BPI to study the kinetics of ciprofloxacin treatment of mice infected with *S. enterica* serovar Typhimurium over the course of 5.5 h. Thanks to the fast turnover of the luciferase enzyme, the effect of the antibiotic could be observed as soon as 1 h post-treatment providing real-time information of the action of ciprofloxacin *in vivo*. Later on, Rocchetta *et al.* (2001) tested the utility of BPI to monitor treatment in a neutropenic mouse thigh model of *E. coli* infection. Although *in vivo* growth of the bioluminescent strain was lower than that of the wild-type strain, a good correlation was found between luminescence and CFUs, except for the earlier time points, with a dose-dependent response for the three antibiotics tested (ceftazidine, tetracycline and ciprofloxacin). More recently, BPI was successfully applied to compare the activity of daptomycin, vancomycin and linezolid in the treatment of peritonitis caused by methicillin-resistant or methicillin-susceptible *S. aureus* in real-time (Mortin *et al.*, 2007). In addition, BPI has been used in two separate studies to determine ceftriaxone and daptomycin efficacy in pneumococcal meningitis (Kadurugamuwa *et al.*, 2005c; Mook-Kanamori *et al.*, 2009;). In the first study, bioluminescence paralleled CFUs in the cerebrospinal fluid, thus providing a much simpler method to monitor infection and treatment. However, in the second work, no luminescence was detected from the majority of the infected mice, which was attributed to the limit of detection of the imaging system.

BPI has also been used to monitor infection by *Mycobacterium bovis* BCG in an immunocompromised mouse model and to evaluate treatment with rifampicin and isoniazid (Heuts *et al.*, 2009). The most notable aspect of this work is that only *luxAB*, the genes for the luciferase, were used because the whole *lux* operon had never been successfully expressed in mycobacteria before. As a result, the authors needed to administer the toxic aldehyde substrate, which was achieved by diluting the substrate in olive oil and ethanol, enhancing its solubility and reducing its toxicity. This allowed Heuts and colleagues to detect infection in the spleens of mice 4 weeks after intravenous inoculation, followed by dissemination to the abdominal cavity. Moreover, a reduction in CFUs and bioluminescence was found 1 and 2 weeks post-antibiotic treatment. Although the researchers proved that *luxAB* can be used to study mycobacterial dissemination, drug efficacy and the role of the immune response, they failed to detect BCG in the lungs of infected mice despite being able to detect bacteria in these organs by colony counting and *ex vivo* imaging. This could be due to a limited distribution of the substrate, which was administered intraperitoneally, thus posing an important limitation to the system as the lungs are of major importance in tuberculosis research. Importantly, the functional expression of the whole *lux* operon in *M. smegmatis* and *M. tuberculosis* has recently been achieved (Andreu *et al.*, 2010), which should prove useful for future *in vivo* imaging studies of mycobacteria.

*In vivo* antibiotic testing is also particularly important and challenging in biofilm infection. The validity of BPI for this purpose was demonstrated in the pioneering work of Kadurugamuwa *et al.* (2003b) who assessed the efficacy of tobramycin, ciprofloxacin and rifampin in the treatment of mice with implanted *S. aureus*-infected catheters. Again, a good correlation was found between CFUs and bioluminescence with the first two antibiotics proving ineffective, while a dose response reduction was obtained after rifampin treatment. Moreover, a greater and faster reduction in bioluminescence was detected compared with CFUs, which is probably related to the fact that protein synthesis is affected before a bactericidal effect can be seen. By monitoring either CFUs or bioluminescence, relapses were detected at different times after finishing the treatment and a large proportion of bacteria recovered from the biofilms were resistant to this antibiotic. Similar results were obtained in subsequent studies in which relapses and resistance after rifampicin treatment were examined in more depth in rifampicin-sensitive (Kadurugamuwa *et al.*, 2004) and rifampicin-resistant *S. aureus* (Yu *et al.*, 2005). Relapses were observed after ciprofloxacin treatment of catheter-associated urinary tract infections of *P. aeruginosa* and *Proteus mirabilis* (Kadurugamuwa *et al.*, 2005b). In this study, catheters were infected both pre- and post-implantation, which resulted in differences in the response to treatment. Moreover, this model allowed monitoring of dissemination of the infection to the upper urinary tract.

One of the main drawbacks of using bioluminescence for such studies is that, because bioluminescence reflects the metabolic state of the microorganism, it is unable to differentiate bactericidal and bacteriostatic treatments. However, this could be inferred by monitoring the time to recovery of a bioluminescent signal after withdrawal of the treatment. If the treatment is bacteriostatic, then the signal could be expected to rapidly reappear once the microorganisms begin to recover. However, if the treatment is bactericidal, the signal would only reappear if not all of the microbial population were killed, and would increase in a manner which reflected the growth of the organism. Furthermore, Brock *et al.* (2008) have suggested that bioluminescence allows researchers to glean important information related to a compound's mode of action. In their studies using *luc*^+^*A. fumigatus*, while the antifungal drugs cycloheximide and nystatin had similar effects on fungal growth, light emission was strongly reduced after treatment with cycloheximide compared with nystatin. In contrast to cycloheximide, which inhibits the *de novo* synthesis of proteins (and hence luciferase expression), nystatin forms ion channels in the fungal membrane. It would appear that while nystatin prevents the growth of the fungus, protein synthesis remains unaffected, at least initially.

Wound infection is another model that has utilized BPI to not only monitor infection, but also treatment (photodynamic therapy) with various degrees of success (Hamblin *et al.*, 2002, 2003; Demidova *et al.*, 2005; Jawhara & Mordon, 2006). Among these works are also two of the few published so far using the firefly luciferase rather than the *lux* operon to study bacterial infection *in vivo* (Jawhara & Mordon, 2004, 2006). Furthermore, BPI has also been used to assess bandage treatment of infected wounds (Burkatovskaya *et al.*, 2006).

While BPI can also be used to assess vaccine efficacy, studies are scant in the literature. However, a good example was reported by Rajashekara *et al.* (2006) using *B. melitensis*. Susceptible and resistant mice independently infected with three attenuated strains of *B. melitensis* carrying the *lux* operon were subsequently challenged with a virulent bioluminescent strain. While all the vaccinated mice survived longer than the unvaccinated ones, the dynamics of persistence and dissemination of the virulent bacteria differed in the susceptible mice depending on the strain used as vaccine. On the contrary, growth of virulent *B. melitensis* was limited to the injection site of resistant mice regardless of the *Brucella* strain used for the vaccination. Therefore, BPI was successfully used to evaluate protection conferred by different vaccine candidates and to determine the effect of host background on the resolution of infection. Other examples include the use of BPI to analyse two potential *Salmonella* vaccine strains in mice of different ages (Burns-Guydish *et al.*, 2007) or to determine how protective antigen immunization affects *B. anthracis* growth and dissemination (Glomski *et al.*, 2007a).

### Monitoring the host response to infection

In addition to studying the pathogen, BPI can be applied to examine the host response during infection. This can be achieved in three ways: (1) using labelled bacteria to infect a mouse strain with an altered immune system (e.g. a knockout mouse lacking a specific aspect of innate or adaptive immunity); (2) using transgenic mice with a reporter gene under the control of a promoter of interest (e.g. that of a cytokine) to study its expression during infection; and (3) a combination of the above two methods, that is, using labelled bacteria to infect transgenic mice.

Most of the examples published to date fall in the first group. For example, BPI was used to study age-related susceptibility to a bioluminescent *S. enterica* serovar Typhimurium derivative (Burns-Guydish *et al.*, 2005). Younger mice were found to be more susceptible to infection, characterized by earlier and greater dissemination. More recently, a *lux*^*+*^*Staphylococcus epidermidis* strain was used to assess the influence of host immune status on device-related infection (Vuong *et al.*, 2008), with immune-compromised mice found to be more susceptible to infection than immune-competent ones. BPI was used as an additional technique to study the innate immune response to *P. aeruginosa* infection in the lungs (Ramphal *et al.*, 2008). Wild-type and TLR2,4^−/−^ mice were infected with wild-type and flagellin-deficient *lux*^*+*^*P. aeruginosa*, and proliferation in the lungs monitored by *in vivo* imaging. The researchers found that the knockout mice were hypersusceptible to infection with the flagellin-deficient *P. aeruginosa* but not wild-type bacteria. This was linked to a failure to control bacterial proliferation in the lungs, which together with other experiments demonstrates the important role of both flagellin and lipopolysaccharides (recognized by TLR2 and 4) in triggering an appropriate innate immunity. Similarly, BPI was applied to monitor bacterial loads in wild-type and knockout mice infected with bioluminescent *C. rodentium* to study the role of MyD88 and the p50 subunit of the transcription factor nuclear factor kappa B (NF-κB) during gastrointestinal infections (Dennis *et al.*, 2008; Gibson *et al.*, 2008;). Both bioluminescence and CFUs were higher in the MyD88^−/−^ mice, while p50^−/−^ mice were found to be unable to clear *C. rodentium*, thus highlighting the significant functions of MyD88 and NF-κB in controlling the infection.

BPI was used to identify the main host receptor involved in sequestration of the murine malaria parasite *P. berghei* (Franke-Fayard *et al.*, 2005), the highly conserved class II scavenger receptor CD36, which is also the main receptor for erythrocytes infected with the human malaria parasite *Plasmodium falciparum*. In an alternative strategy, Amante *et al.* (2007) administered anti-CD25 monoclonal antibody to mice in order to deplete regulatory T cells and utilized BPI to follow the dissemination of *P. berghei*. Interestingly, treatment was found to protect the animals from experimental cerebral malaria, with a reduction in the accumulation of parasites in the vasculature and brain in comparison with control animals. This report was the first example to show that regulatory T cells can contribute to pathogenesis during infectious disease by suppressing antiparasitic immunity.

Interferons are one of the key mediators of host innate immunity to viral infection, inducing an antiviral state in infected cells and regulating the adaptive immune response to viruses. Type I interferons are secreted by most cells in response to viral infection, while the production of type II interferon is restricted to activated T cells, natural killer cells, natural killer T cells and dendritic cells. Luker *et al.* (2003) utilized BPI to determine the effects of type I and II interferons on the replication and tropism of bioluminescent HSV-1 in mice and demonstrated differing effects of the two interferon types in limiting systemic viral dissemination. Following footpad or ocular infection of mice lacking type I interferon receptors, HSV-1 spread to the parenchymal organs, including the lungs, liver, spleen and regional lymph nodes, but mice survived. In contrast, deletion of both type I and II interferon receptors produced widespread viral dissemination to visceral organs and the nervous system, which was invariably lethal. Furthermore, type II receptor knockout and wild-type mice had comparable viral replication and localization, with no systemic viral spread or lethality. This led the authors to conclude that while isolated deficiency of type II interferon receptors did not affect HSV-1 pathogenesis, loss of these receptors in combination with genetic deletion of type I receptors has a profound effect on susceptibility to HSV-1. Subsequently, Luker *et al.* (2005) demonstrated that the replication of firefly luciferase-expressing vaccinia virus was significantly greater in mice lacking receptors for type I interferon compared with wild-type mice, and that knockout mice had greater viral dissemination to the liver and spleen.

The second approach of using transgenic reporter mice has not yet been fully exploited in infectious diseases research, despite the availability of a large number of these models. For example, transgenic mice with the firefly luciferase gene under the control of the promoter of either iNOS (Zhang *et al.*, 2003), NF-κB (Carlsen *et al.*, 2002) or IL-1β (Li *et al.*, 2008) have been developed. In these models, luminescence has been shown to respond to inflammatory signals, such as exposure to lipopolysaccharide, suggesting they should prove useful in studying the host's response to infection. Indeed, Hemrajani *et al.* (2008) utilized an NF-κB-reporter mouse to investigate the interplay between a secreted bacterial virulence factor NleH, and the immune system in *C. rodentium* infection. The researchers found that the reporter animals expressed high basal levels of luciferase from a number of organs, which resulted in no differences being detectable by live imaging of reporter animals infected with wild-type and *nleH-*deficient *C. rodentium*. However, when the colons of infected and uninfected reporter animals were imaged *ex vivo*, the bioluminescent signal was significantly higher in mice infected with the wild-type *C. rodentium* compared with those inoculated with the NleH mutant, and similar to the level of signal in mice inoculated with tumour necrosis factor-α as a positive control.

Because transgenic reporter mice utilize the firefly luciferase, it is possible to monitor infections with *lux*^*+*^ bacteria within the same animal, given that the spectra for the bacterial and firefly luciferase are different, and the firefly luciferase produces light only when the substrate luciferin is provided. This combined approach was intelligently applied by Kadurugamuwa *et al.* (2005a) to concurrently track infection by *S. pneumoniae* and the associated neuronal damage. Progression of the infection from the site of inoculation, the subarachnoid cistern in the brain, down through the spinal cord could be monitored by imaging the luminescence produced by the *lux*^*+*^*S. pneumoniae*. The host response, tracked as glial fibrillary acidic protein (a marker of neuronal damage) induced expression of the firefly luciferase, proceeded in a parallel manner. This model was also used to evaluate the efficacy of antibiotic treatment in eradicating infection and limiting the neuronal damage responsible for the sequelae associated to bacterial meningitis. The researchers found that although therapy successfully eliminated the infection even when the treatment was initiated 17 h postinfection, neuronal injury was completely prevented only when the treatment started 11 h postinfection, thus emphasizing the importance of prompt treatment.

## Noninvasive imaging of fluorescence in infectious disease research

Imaging fluorescently labelled microorganisms noninvasively *in vivo* is still in its infancy and reports in the literature are scant (Zhao *et al.*, 2001; Mehta *et al.*, 2008; Kong *et al.*, 2009;). This is largely due to the success with which microorganisms can be labelled with bioluminescence coupled with a lack of fluorescent reporters with suitable emission wavelengths. Indeed, almost all the published reports utilize GFP, despite its unsuitable excitation and emission wavelengths. However, the relative explosion in the number of red-shifted FPs developed in recent years should see this exciting field now begin to flourish.

Mehta *et al.* (2008) used BPI to visualize a GFP-expressing derivative of the parasite *L. amazonensis* in a mouse foot-pad model. This infection model has the advantage of utilizing a superficial site that is well-isolated from the rest of the body; therefore the background fluorescence is relatively low while light penetration is high. Classically, parasite burden and progression of the infection is monitored by measuring the thickness of the foot pad with a calliper (Courret *et al.*, 2003). The authors were able to demonstrate that parasite burden could be reliably measured using BPI by correlating fluorescence intensity to parasite numbers, and that this approach was in fact more sensitive than the calliper method. Parasites could be visualized 1 week after inoculation with 10^7^ organisms while thickness of the foot pad only increased significantly approximately 3 weeks postinfection. Furthermore, after 3 weeks of immunotherapy with the Leish 111f+MPL-SE vaccine the infection was reduced; significant differences between the treated and control group were observed after 14 and 35 days when using imaging vs. calliper measurement, respectively. Zhao *et al.* (2001, 2005b, 2007) have visualized a GFP-expressing auxotroph of *S. enteric* serovar Typhimurium in numerous different tissues. Interestingly, their work has not involved studying the pathogenicity mechanisms of *Salmonella* but has focussed on utilizing the GFP-expressing strain as an effective therapy for cancer.

Recently, advances in the development of NIR probes have been exploited to visualize schistosomes *in vivo*. Schistosomes are intravascular, parasitic helminths that have a high concentration of cathepsin in their gut and can therefore activate the NIR probe ProSense680 (VisEn Medical Inc., Bedford, MA) (Krautz-Peterson *et al.*, 2009). To label the intravascular worms, infected mice were simply injected intravenously with the probe, leading to strong fluorescence throughout the intestines. Five weeks after infection, the authors could visualize as few as three worms using FMT and reported that the main accumulation site for *Schistosoma mansoni* is the upper mesenteric veins of the abdomen. The fluorescent signal correlated strongly with parasite numbers and treatment with the drug praziquantel significantly reduced the fluorescent signal when compared with untreated controls.

Another exciting strategy utilizing NIR probes was recently reported by Kong *et al.* (2010). Termed reporter enzyme fluorescence, this elegant strategy involves combining endogenous microbial enzymes with custom-designed fluorogenic substrates. Importantly, the fluorogenic substrates are linked to a quencher molecule and hence only become fluorescent after interacting with the target enzyme. Furthermore, use of a natural enzyme prevents the potential for unforeseen metabolic impacts due to the expression of heterologous genes. Perhaps most exciting, REF has the potential to allow the detection of microorganisms in patients as it does not rely on the detection of genetically modified organisms. Kong and colleagues utilized β-lactamase, a naturally occurring enzyme expressed by numerous bacteria, but not their eukaryotic hosts, and which confers resistance to penicillin and cephalosporin antibiotics (Davies, 1994). The *M. tuberculosis*β-lactamase, BlaC, is located on the bacterial surface (Flores *et al.*, 2005) and constitutively expressed *in vivo* (Talaat *et al.*, 2004). Using REF, the researchers were able to detect approximately 10^2^ CFU of *M. tuberculosis, P. aureuginosa* and *S. aureus in vitro* and as few as 10^4^ CFU of *M. tuberculosis* in the lungs of living mice (Kong *et al.*, 2010).

## Future perspectives

### Dual bioluminescent/fluorescent labelling of microorganisms

Providing the burden of expression is not too great, dual labelling of microorganisms with fluorescent and bioluminescent reporter genes takes advantage of the differential behaviour and cofactor requirements of FPs and luciferases, thus combining the strengths of each system. For example, various groups have described strains marked with a luciferase in tandem with GFP (Unge *et al.*, 1999; Qazi *et al.*, 2001; Tamagnini *et al.*, 2008;), which has allowed the discrimination between microbial counts (by fluorescence) and metabolic activity (by bioluminescence). Furthermore, fluorescence labeling allows samples to be analysed by fluorescence microscopy and flow cytometry in addition to BPI. Importantly, this means that more data can be gathered using fewer experimental animals while also bridging the gulf between the macroscopic and microscopic levels of resolution, that is, individual microorganisms at one end of the imaging spectrum and the detection of mass populations in specific niches in the living animal at the other.

### Spectral unmixing

The increasing availability of defined bandwidth filters for capturing photonic signals, and improvements in spectral unmixing algorithms to differentiate between signals with different emission spectra, opens up exciting new avenues for infectious disease research. One such avenue is the ability to simultaneously monitor different bacterial strains within the same animal, for example, in competition experiments between wild-type and mutant strains of the same organism, or to follow the dynamic inter-relationships between different species. In order not to impose additional and/or differing metabolic burdens on the bacteria under study, the reporter genes would ideally be extremely similar to allow true comparisons to be made between strains. Branchini *et al.* (2007) have previously described the development of two thermostable firefly luciferase variants (PpyRE-TS and PpyGR-TS). The variants, which differ by only four amino acids have readily distinguishable emission spectra, with peaks of 612 nm for PpyRE-TS and 552 nm for PpyGR-TS. Foucault *et al.* (2010) recently demonstrated for the first time the use of these reporters and spectral unmixing to simultaneously monitor two populations of *E. coli* in the mouse gastrointestinal tract.

### Multimodality imaging

The ability to coregister optical-imaging data with other imaging modalities such as magnetic resonance imaging (MRI) (Medarova *et al.*, 2009), positron emission tomography (PET) (Cao *et al.*, 2006), single photon emission computed tomography (SPECT) and X-ray computed tomography (CT) is an exciting development, allowing each modality to bring its unique advantages to the fore, while providing complementary information. Indeed, the ability to monitor pathogen dynamics and host gene expression by optical imaging, and the resultant changes in host physiology and anatomy using other imaging modalities, will considerably enhance our understanding of the complexities of infection processes *in vivo*. Furthermore, the development of multiple probes is attracting increasing attention. Brader *et al.* (2008) recently reported the visualization in mice of a bioluminescent strain of *E. coli* by both optical imaging and PET (based on the expression of endogenous bacterial thymidine kinase) while two commercial optical-X-ray CT imaging systems are now available (Table 2).

### BPI in nonanaesthetized animals

In planar imaging, the time required to capture an image is determined by the level of photonic signal and the sensitivity of the detection system, and is often in the region of 30 s to 5 min. For this reason, mice are generally anaesthetized for restraint purposes. However, a number of researchers have highlighted the need for caution when using anaesthesia in animal models. There are reports in the literature that anaesthetic agents can influence neutrophil and monocyte function (Welch, 1984); even a very brief exposure (<1 min) to isoflurane can profoundly affect proinflammatory parameters in experimental endotoxaemia in rats (Hofstetter *et al.*, 2005), and that a number of anaesthetic agents can inhibit the growth of some microorganisms but not others (Molliex *et al.*, 1998; Asehnoune *et al.*, 2000; Karabiyik *et al.*, 2007;). Importantly, it was noted many years ago that the presence of relatively low levels of general anaesthetics inhibited the light emitted by cultures of various bioluminescent bacteria (Johnson *et al.*, 1951). Subsequently, it was shown that a good correlation existed between the concentrations required to reduce light output from these bacteria and those required to induce anaesthesia in animals (Halsey & Smith, 1970; White & Dundas, 1970;). More recently, the activity of purified firefly luciferase has been shown to be inhibited by a diverse range of general anaesthetics with a sensitivity that closely parallels anaesthetic potencies in animals over five orders of magnitude (Franks & Lieb, 1984). With the absolute requirement for anaesthesia to perform the experiments, there has been no means of investigating the extent to which anaesthesia may influence optical-imaging studies. Indeed, researchers have been limited to comparisons of different anaesthetic agents (Cui *et al.*, 2008). Our own preliminary data suggest a 10-fold increase in signal intensity when using the inhalational anaesthetic isoflurane compared with an intraperitoneal injection of ketamine and xylazine to image bioluminescent *C. rodentium* in the gastrointestinal tract of infected mice (Wiles, 2010). However the opposite may be true for imaging bioluminescent pathogens in the lungs. Recently, optical-imaging systems using electron-multiplying CCD cameras have become commercially available (Table 2). In essence, this means that photonic signals can now be captured within milliseconds, allowing data to be acquired on nonanaesthetized animals. This is a significant and exciting advance that will allow researchers to investigate biological systems using optical imaging without the influence of anaesthesia, as well as offering the obvious important benefits to animal welfare.

## Conclusions

The use of BPI is becoming widespread in infectious disease research for several reasons: first, the novel insights the technique can provide into microbial behaviour and the niches exploited by microorganisms *in vivo*; second, the impact of the technique on two of the guiding principles of using animals in research, namely refinement and reduction. Indeed, it is not unreasonable to speculate that the financial benefits of acquiring maximal information using fewer animals will see the use of BPI continue to grow in the current economic climate. Furthermore, advances in hardware, software and reporter development will continue to expand the range of experimental questions that can be addressed using BPI.

## Note added in proof

In an exciting development, Close *et al.* (2010) have recently reported the expression of a synthetic codon-optimized lux operon in a mammalian HEK293 cell line *in vitro* and *in vivo*.

## Statement

Re-use of this article is permitted in accordance with the Terms and Conditions set out at http://wileyonlinelibrary.com/onlineopen#OnlineOpen_Terms
